# A 3D atlas of the trigeminal nerve and its relevance for comparative studies of the masticatory apparatus in rodents

**DOI:** 10.1111/joa.70212

**Published:** 2026-07-13

**Authors:** Lionel Hautier, Marcos Angelo Alves, Léa Da Cunha, Benoit Moison, Ludovic Besson, Pierre‐Henri Fabre

**Affiliations:** ^1^ ISEM, Univ Montpellier, CNRS, IRD Montpellier France; ^2^ Mammal Section, Life Sciences, Vertebrate Division The Natural History Museum London UK; ^3^ Programa de Pós‐Graduação em Sistemática, Taxonomia Animal e Biodiversidade, Museu de Zoologia da Universidade de São Paulo São Paulo Brazil; ^4^ Muséum d'histoire Naturelle Gabriel Foucher, Les Rives d'Auron Bourges France; ^5^ Institut Universitaire de France Paris France; ^6^ Department of Mammalogy, Division of Vertebrate Zoology American Museum of Natural History New York USA

**Keywords:** comparative anatomy, masticatory muscles, rodents, trigeminal nerve

## Abstract

The trigeminal nerve (CN V) is a key cranial nerve responsible for sensory input from the face and motor control of the masticatory muscles, playing a central role in mandibular movement and oral cavity function across tetrapods. Despite its importance and its link with the masticatory musculature, no comprehensive comparative study of the trigeminal nerve across rodent groups has yet been conducted. This is partly due to the difficulty of visualizing nerves using conventional dissection techniques, and partly due to the complex, three‐dimensional organization of the nervous system. Here, we combined diffusible iodine‐based contrast‐enhanced CT imaging (diceCT) with traditional dissection to characterize the trigeminal nerve and its relationship to masticatory muscles in three representative rodents: the rat, the red squirrel, and the Guinea pig. Our findings demonstrate that the broad architecture of the nervous system is highly constrained but exhibits fine‐scale plasticity in nerve branching and muscle innervation. While the gross nerve organization is largely conserved, we identified species‐specific differences, particularly in Guinea pigs, linked to skeletal and muscular modifications. The study underscores the value of integrating 3D imaging with dissection to accurately capture nerve morphology and discuss muscle homologies, with implications for comparative anatomy, functional morphology, and paleontological reconstruction.

## Graphical abstract

The trigeminal nerve of the rat (*Rattus norvegicus*) and its relationship with different masticatory muscles. dig, digastric nerve; ep, external pterygoid nerve; ip, internal pterygoid nerve; maadm, branch of the masseteric nerve for the anterior deep masseter; mapdm, branches of the masseteric nerve for the posterior deep masseter; masm, branch of the masseteric nerve for the superficial masseter; mazyg, branch of the masseteric nerve for the zygomaticomandibularis; mh, mylohyoid nerve; mta, medial temporal anterior nerve; mtp, medial temporal posterior nerve; V3, mandibular division.

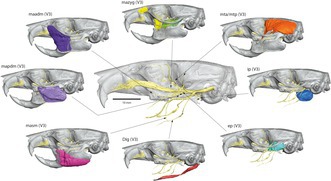

## 1 INTRODUCTION

**FIGURE 1:**
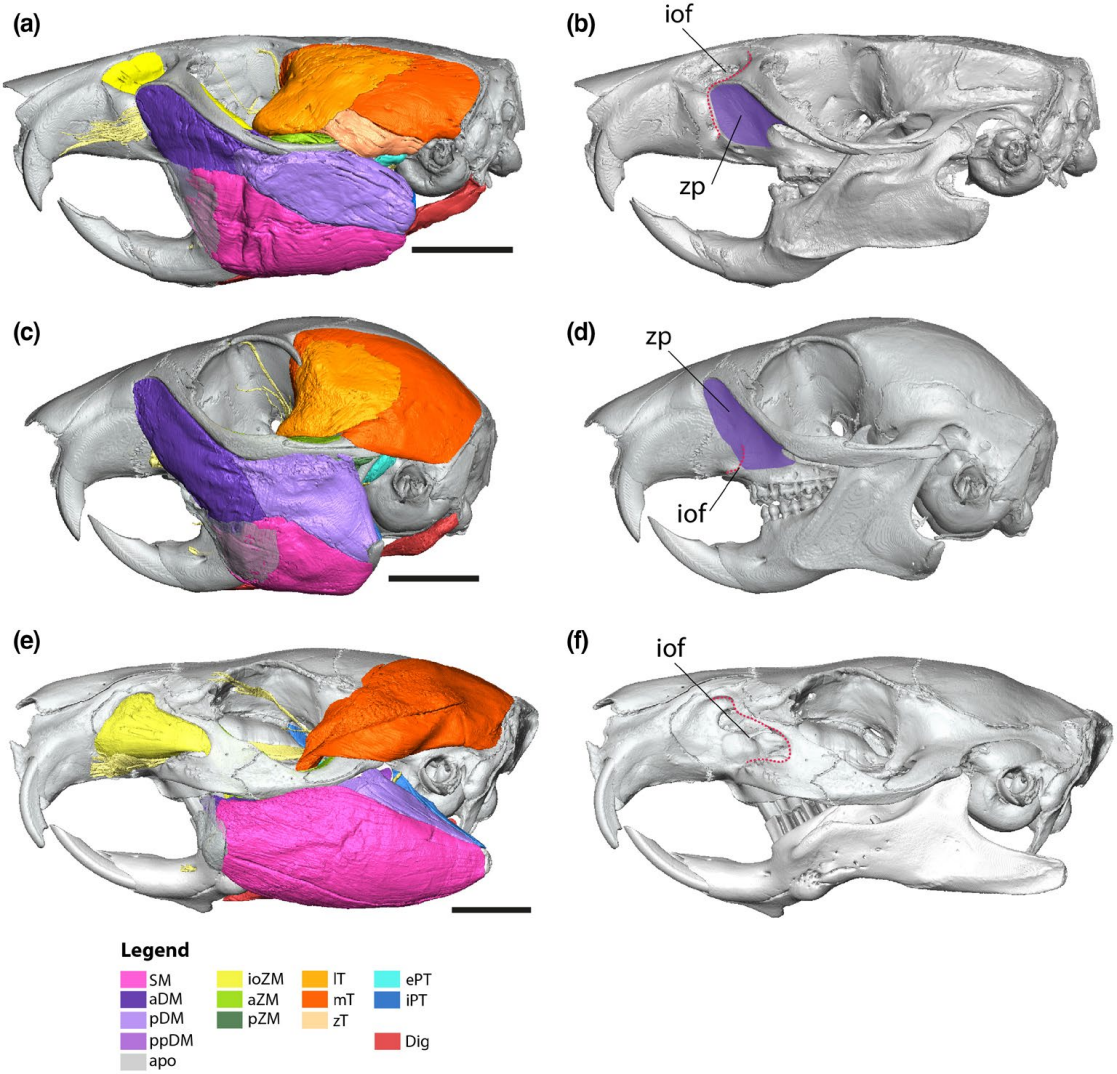
Left lateral view of the 3D reconstruction of the masticatory musculature, trigeminal nerve (pale yellow), and skull of (a, b) the brown rat *Rattus norvegicus*; (c, d) the red squirrel *Sciurus vulgaris*; and (e, f) the Guinea pig *Cavia porcellus*. The right column (b, d, and f) illustrates the morphological characters traditionally used to define rodent cranial morphotypes (myomorphy, sciuromorphy, and hystricomorphy) with the zygomatic plate (in violet, the origin of *aDM*) and the infra‐orbital anterolateral edge of the maxillary mallar process (red dot lines, origin of the *IOZM* in myomorphs and hystricomorphs). *aDM*, anterior deep masseter; *aZM*, anterior part of the zygomaticomandibular muscle; *apo*, aponeurosis; *Dig*, digastric muscle; *ePT*, external pterygoid muscle; *iof*, infraorbital foramen; *iPT*, internal pterygoid muscle; *lT*, lateral part of the temporal muscle; *mT*, medial part of the temporal muscle; *ioZM*, infraorbital part of the zygomaticomandibular muscle; *pDM*, posterior deep masseter muscle; *ppDM*, posterior portion of the posterior deep masseter muscle (only present in *Cavia*); *pZM*, posterior part of the zygomaticomandibular muscle; *SM*, superficial masseter muscle; *TM*, transverse mandibular; *zp*, zygomatic plate; *zT*, zygomatic part of the temporal muscle. Scale bars are 10 mm.

Rodents represent one of the most diverse mammalian groups, yet their cranial architecture exhibits a remarkably limited set of morphotypes associated with the reinforcement of anteroposterior mandibular movements used during gnawing (Brandt, 1855; Cox & Jeffery, 2011; Hautier et al., 2008; Hautier & Cox, 2015; Waterhouse, 1839a, 1839b, 1842a, 1842b, 1848; Wood, 1965). Strong functional constraints related to mastication appear to have limited the range of possible evolutionary pathways, promoting repeated convergence in cranial morphology and thereby complicating attempts to resolve intra‐ordinal relationships (Hautier et al., 2012). The primitive cranial condition, that is, protrogomorphy, is only found in a single extant species (*Aplodontia rufa* [Rafinesque, 1817], the mountain beaver; Druzinsky et al., 2011), although it is well documented among Paleogene rodents (Vianey‐Liaud & Marivaux, 2021). Three derived morphotypes evolved from this condition: sciuromorphy, hystricomorphy, and myomorphy. These morphotypes differ primarily in the anterior origins of the masticatory musculature and are generally associated with distinct feeding behaviors and dietary specializations (Figure 1; Cox et al., 2012; Cox & Jeffery, 2011; Wood, 1965).

Although cranial and dental morphology have been extensively studied, the soft tissues of the masticatory system remain comparatively understudied. Despite the longstanding use of muscular characteristics in rodent systematics, detailed characterizations of the jaw muscles are available for only a limited portion of extant rodent diversity (e.g., Baverstock et al., 2013; Cox et al., 2020; Cox & Baverstock, 2016; Cox & Faulkes, 2014; Da Cunha et al., 2024; Druzinsky, 2010; Fabre et al., 2017; Ginot et al., 2018; Hautier, 2010; Hautier & Saksiri, 2009; Klingener, 1964; Parsons, 1894; Potapova, 2020; Tullberg, 1899). Furthermore, the use of heterogeneous terminologies across studies has hindered the identification of homologous structures at the scale of the entire order (Druzinsky et al., 2011, 2016). These gaps become even more apparent when considering the associated nervous system. Although several authors have proposed classifying masticatory muscles based on their innervation in mammals (Druzinsky et al., 2016; Nakajima & Townsend, 2009; Shimokawa et al., 1998; Tomo et al., 1993), innervation patterns in rodents have rarely been examined in detail alongside anatomical descriptions of the masticatory apparatus (Greene, 1935).

The trigeminal nerve, also known as the fifth cranial nerve (CN V), is a crucial component of the mammalian nervous system responsible for transmitting senso‐motory information from the face to the brain, including touch, pain, temperature sensation, as well as motor functions of the facial and masticatory muscles (Evans & De Lahunta, 2012; Fillmore & Seifert, 2015; Greene, 1935). As its name implies, this nerve is composed of three major branches: the ophthalmic (V1), maxillary (V2), and mandibular (V3) divisions. The ophthalmic and maxillary divisions are entirely sensory, while the mandibular one serves both sensory and motor functions (Evans & De Lahunta, 2012; Fillmore & Seifert, 2015; Greene, 1935). The motor branches of the mandibular division innervate the masseter, the temporal, the pterygoid, and the mylohyoid muscles, as well as the anterior belly of the digastric muscle (Evans & De Lahunta, 2012; Fillmore & Seifert, 2015; Greene, 1935). As such, this single cranial nerve is solely responsible for innervating the muscles governing mandibular movement and changes in oral cavity volume, highlighting its fundamental role during mammal evolution. Yet, despite this central role, no comprehensive comparative anatomical study of the trigeminal nervous system across rodent groups has been conducted.

Murine models (i.e., mice and rats) have been extensively used to explore the functional role of the trigeminal nerve (Dingle et al., 2019; Hadlock et al., 2007; Skouras & Angelov, 2010), primarily in studies aimed at understanding nerve regrowth and the restoration of masticatory and facial muscle function following injury‐induced paralysis (Hadlock et al., 2008, 2010; Waite, 1984). However, aside from Greene's (1935) seminal anatomical work on the rat, detailed descriptions of trigeminal innervation patterns across rodents remain limited. The inherent difficulty in visualizing nerves during conventional dissection, particularly in fixed and alcohol‐preserved specimens, undoubtedly contributes to this challenge, as does the complex three‐dimensional organization of the nervous system. High‐resolution CT scanning methods have proven to be a powerful tool to unravel intricate anatomical details, and much headway has been made recently to describe the mammalian masticatory musculature with diffusible iodine‐based contrast‐enhanced computed tomography (diceCT, e.g., Da Cunha et al., 2024; Gignac et al., 2016; Sharp & Trusler, 2015). Although the brain and its endocast have received considerable attention owing to advances in X‐ray microtomography (Bertrand et al., 2017, 2018; Bertrand & Silcox, 2016), nerves remain comparatively understudied within the nervous system.

Using this 3D techniques, alongside conventional dissections, we propose to explore the morphological variation of the trigeminal nerve in three representative species from the main rodent lineages: *Rattus norvegicus* (Berkenhout, 1769) (the brown rat, representing the myomorphous condition observed in most members of the mouse‐related clade); *Sciurus vulgaris* Linnaeus, 1758 (the red squirrel, representing the squirrel‐related clade and the sciuromorphous condition); and *Cavia porcellus* (Linnaeus, 1758) (the Guinea pig, representing the hystricomorphous condition observed in Hystricomorpha). Here, we will particularly focus on describing the anatomy of the trigeminal nerve and its association with masticatory musculature, aiming to provide further clues to homologize muscular parts across the entire order.

## 2 MATERIALS AND METHODS

### 2.1 Data acquisition, dissection and processing

**FIGURE 2:**
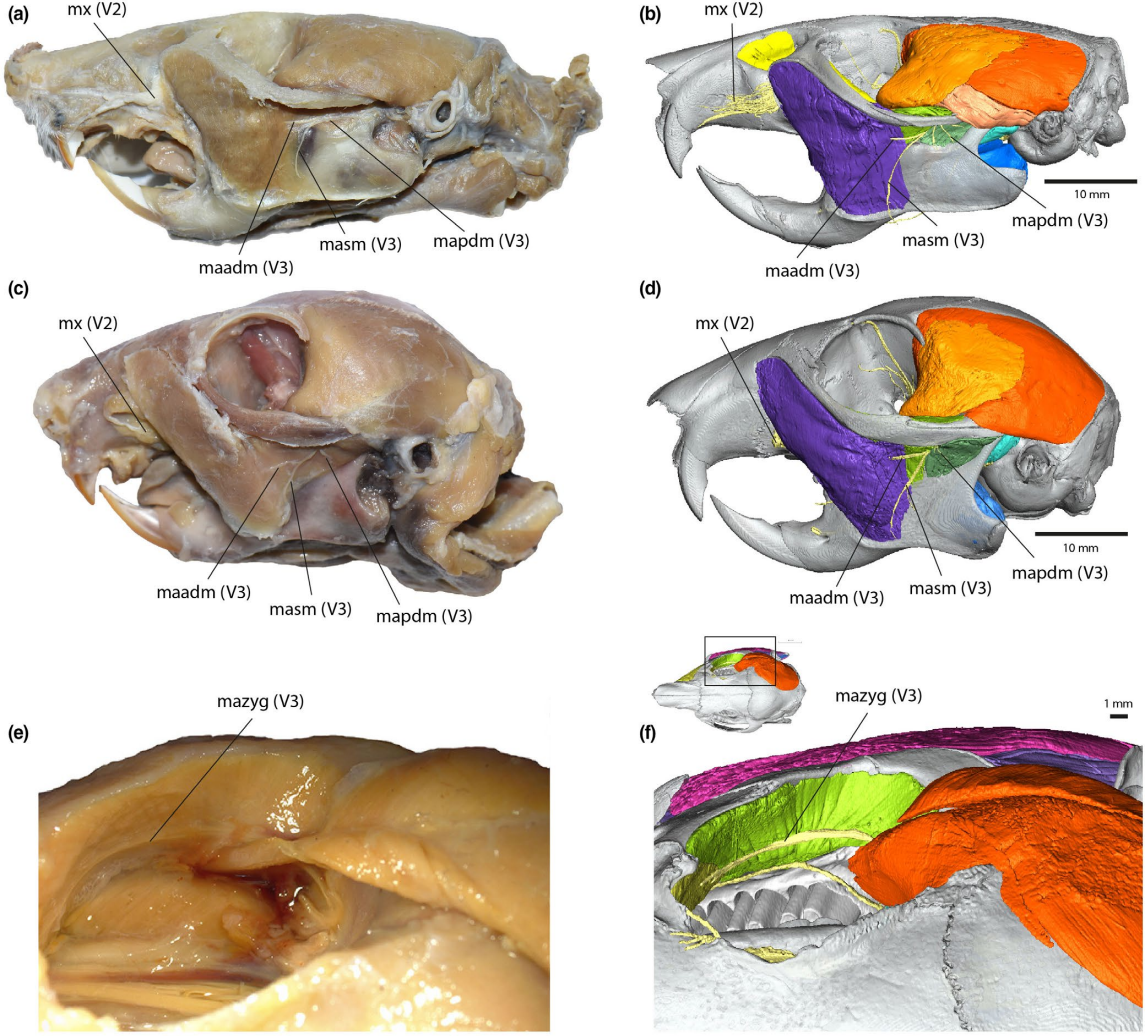
Comparison of dissection methods: Traditional (a, c, and e) and virtual (b, d, and f) dissections of *Rattus norvegicus* (lateral view; a and b), *Sciurus vulgaris* (lateral view; c and d), and *Cavia porcellus* (dorsal view of the orbital region; e and f). *maadm*, branch of the masseteric nerve for the anterior deep masseter muscle; *mapdm*, branch of the masseteric nerve for the posterior deep masseter muscle; *masm*, branch of the masseteric nerve for the superficial masseter muscle; *mazyg*, branch of the masseteric nerve for the zygomaticomandibular muscle; *mx*, maxillary nerve; V2, maxillary division; V3, mandibular division.

Dissections were performed on adult specimens of three rodent species, *Rattus norvegicus* (*n* = 1; UM‐ZOOL‐3105), *Sciurus vulgaris* (*n* = 4; MNHN ZM‐MO 13269, BOUM‐2022.2.12, BOUM‐2022.2.15, and BOUM‐2023.2.14), and *Cavia porcellus* (*n* = 2; UM‐ZOOL‐499V and UM‐ZOOL‐572N). These specimens belong to the collection of the University of Montpellier (UM), of the Natural History Museum Gabriel Foucher of Bourges (BOUM), and of the Natural History Museum in Paris (MNHN). They were initially fixed in 10% formalin before being preserved in 70% ethanol. Traditional dissections were performed on one side of the specimen under a Leica S9i stereomicroscope equipped with ×10 eyepieces to document the anatomy of the trigeminal nerve and masticatory muscles (Figure 2). Each dissection step was carefully photographed using the integrated 10MP digital camera of the stereomicroscope as well as a Nikon D750 with a AF‐S Micro Nikkor 105 mm 1:2.8G ED lens mounted on a copy stand to capture the overall innervation and muscle patterns of the specimens.

### 2.2 Nomenclature and anatomical descriptions

Due to the wide variation in craniomandibular morphology and the different methods used to define muscle anatomy in rodents and mammals as a whole, there are significant discrepancies in the nomenclature of masticatory muscles in the literature (Druzinsky et al., 2011). This study follows the nomenclature used by Da Cunha et al. (2024) for muscle parts (Figure 1). The only difference is the recognition of a zygomatic part of the temporal muscle in *Rattus norvegicus*. Recognition of individual muscles was primarily based on their areas of origin and insertion on the skull and mandible, respectively, but the relative position, orientation of muscle fibers and innervation pattern of the trigeminal nerve were used to distinguish parts of different muscle groups. Unlike the masticatory musculature, the nerves of rodents have been poorly described. Here, we follow the nomenclature proposed by Greene (1935). Due to the limited number of species, we chose to provide a thorough anatomical description of the innervation patterns in the rat and a comparative description for the squirrel and the Guinea pig.

### 2.3 Three‐dimensional (3D) imaging and reconstruction

The best preserved individuals from each species were selected for diceCT, a method of contrast‐enhanced micro‐computed tomography (microCT) using Lugol's iodine (I_2_KI). This method allows nondestructive visualization of nerve and muscle configuration, allowing comparison with observations made during traditional dissection (Figure 1). Lugol's iodine was chosen as the staining agent due to its cost‐effectiveness, excellent resolution of muscle fibers and efficacy on formalin‐fixed specimens (Gignac et al., 2016). After one side had been dissected, the specimens were placed in sealed containers filled with a 5% I_2_KI solution, with sufficient volume to facilitate the diffusion process. The duration of staining varied depending on the size of the sample: *C. porcellus* (UM‐ZOOL‐499V) required 3 weeks, while *R. norvegicus* (UM‐ZOOL‐3105) and *S. vulgaris* (BOUM‐2022.2.12) required 2 weeks. Each specimen was scanned twice—before and after contrast enhancement—on an EasyTom 150 X‐ray microtomograph at the *Institut des Sciences de l'Évolution de Montpellier*, University of Montpellier (MRI; ISE‐M, Montpellier, France). The voxel sizes were as follows: *Rattus*—soft tissue, 0.0176981 μm; bones, 0.0167528 μm. *Cavia*—soft tissue, 0.0300039 μm; bones, 0.0238193 μm. *Sciurus*—soft tissue, 0.0239901 μm; bones, 0.0239867 μm. Independent imaging of bone and muscle facilitated skull segmentation from bone‐optimized scans and automatic alignment with muscle‐optimized scans using the “Magic Wand” tool and the “Register Images” module of the Avizo software (Thermo Fisher Scientific), respectively. Manual segmentation was performed for nerves due to their size and minimal gray scale variation compared to muscle fibers (Figure S1). Aponeuroses and tendons were distinguished from muscle fascicles, and were also manually segmented where visible in the diceCT scans. Muscle segmentation was performed incrementally every 10 slices for *Rattus* and *Sciurus* and every 20 slices for *Cavia*. Image data and pre‐segmented muscle slices were imported into Biomedisa (Lösel et al., 2020) with the “all axes” parameter selected in the label field. The final segmentation was obtained by selecting the cleaned and smoothed parameters, with minor adjustments to correct errors from the semi‐automatic image segmentation. Three‐dimensional surface models of bone and muscle were generated and downsampled to reduce file size and facilitate 3D visualization. They are available on MorphoMuseuM (Hautier et al., 2026).

## 3 RESULTS

**FIGURE 3:**
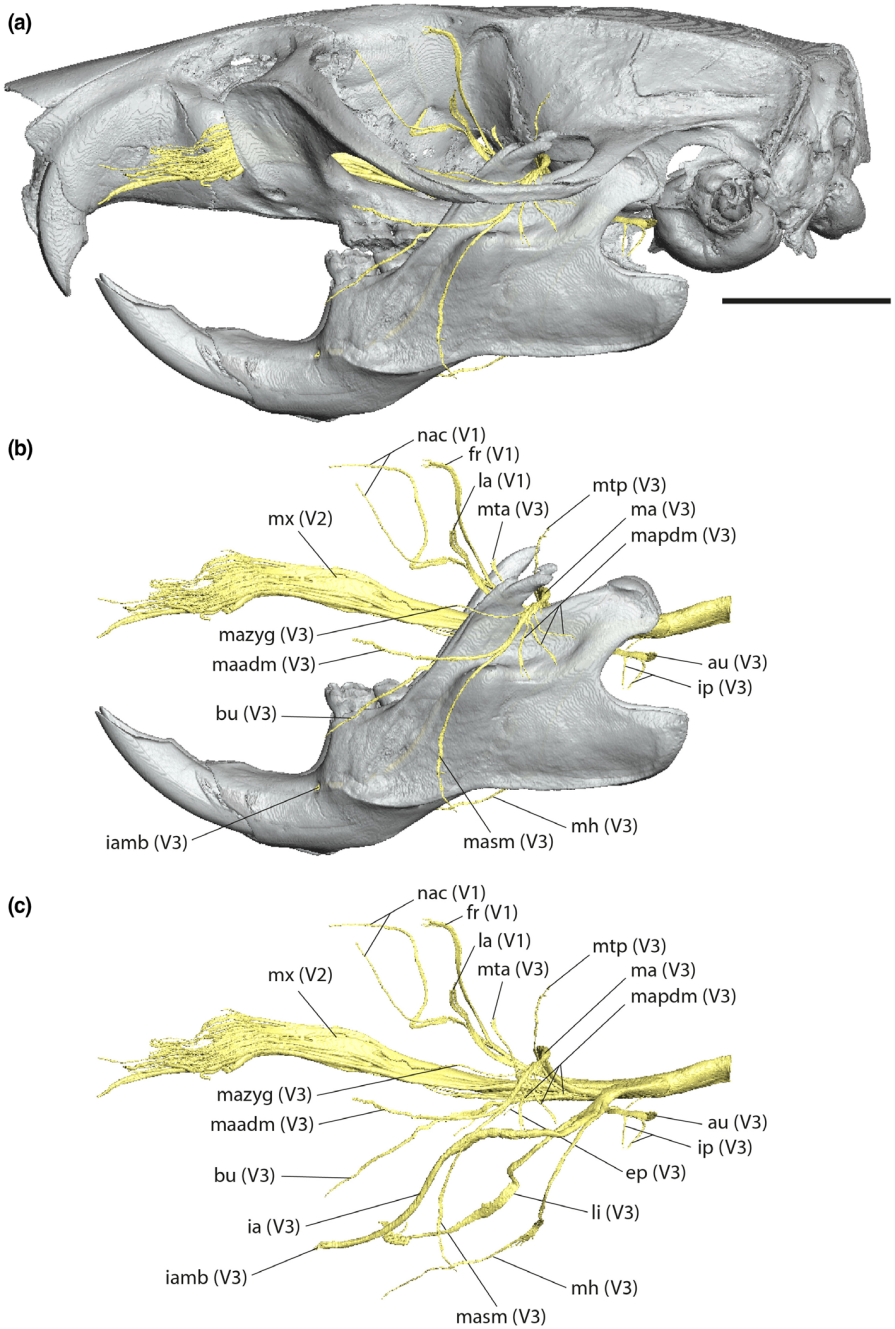
Virtual dissection of the trigeminal nerve of *Rattus norvegicus* in lateral view: (a), with the cranium and the mandible; (b), with the mandible only; (c), without the cranium and the mandible. *au*, auriculotemporal nerve; *bu*, buccinator nerve; *ep*, external pterygoid nerve; *fr*, frontal nerve; *iamb*, mental branch of inferior alveolar nerve; *ia*, inferior alveolar nerve; *ip*, internal pterygoid nerve; *la*, lacrimal nerve; *li*, lingual nerve; *ma*, masseteric nerve; *maadm*, branch of the masseteric nerve for the anterior deep masseter muscle; *mapdm*, branches of the masseteric nerve for the posterior deep masseter muscle; *masm*, branch of the masseteric nerve for the superficial masseter muscle; *mazyg*, branch of the masseteric nerve for the zygomaticomandibular muscle; *mh*, mylohyoid nerve; *mta*, medial temporal anterior nerve; *mtp*, medial temporal posterior nerve; *mx*, maxillary nerve; *nac*, nasociliary nerve; V1, ophthalmic division; V2, maxillary division; V3, mandibular division. Scale bars are 10 mm.

**FIGURE 4:**
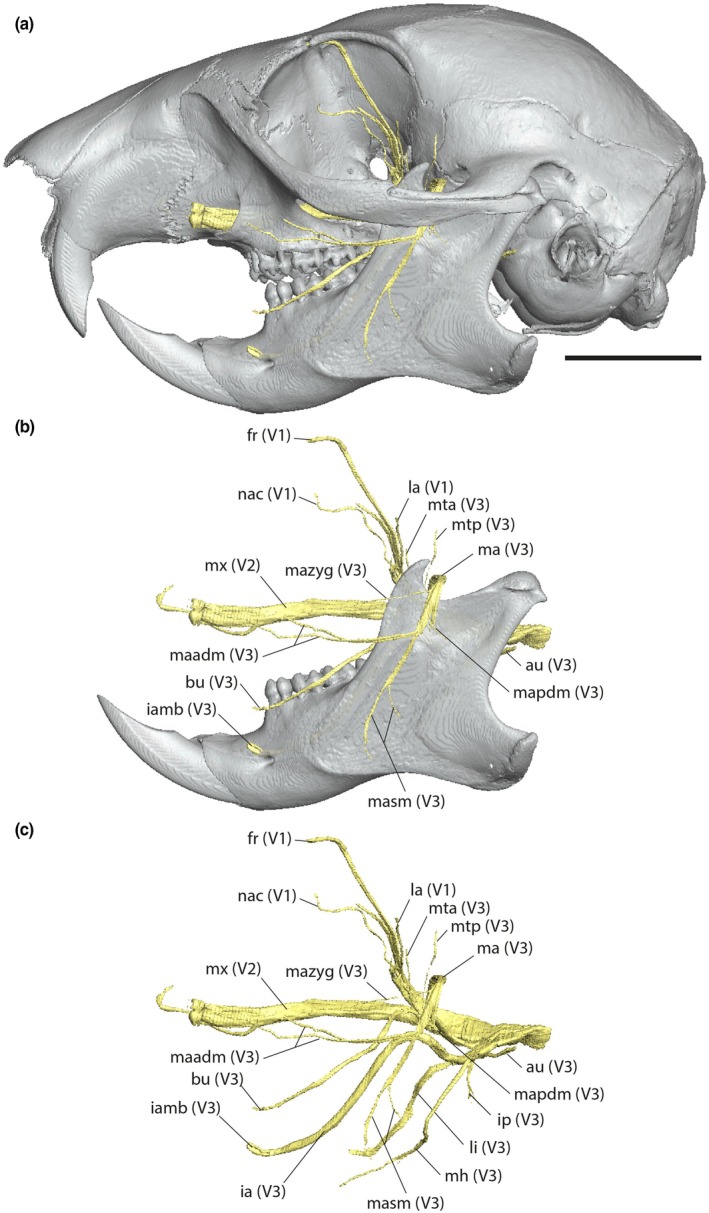
Virtual dissection of the trigeminal nerve of *Sciurus vulgaris* in lateral view: (a), with the cranium and the mandible; (b), with the mandible only; (c), without the cranium and the mandible. *au*, auriculotemporal nerve; *bu*, buccinator nerve; *fr*, frontal nerve; *iamb*, mental branch of inferior alveolar nerve; *ia*, inferior alveolar nerve; *ip*, internal pterygoid nerve; *la*, lacrimal nerve; *li*, lingual nerve; *ma*, masseteric nerve; *maadm*, branch of the masseteric nerve for the anterior deep masseter muscle; *mapdm*, branches of the masseteric nerve for the posterior deep masseter muscle; *masm*, branch of the masseteric nerve for the superficial masseter muscle; *mazyg*, branch of the masseteric nerve for the zygomaticomandibular muscle; *mh*, mylohyoid nerve; *mta*, medial temporal anterior nerve; *mtp*, medial temporal posterior nerve; *mx*, maxillary nerve; *nac*, nasociliary nerve; V1, ophthalmic division; V2, maxillary division; V3, mandibular division. Scale bars are 10 mm.

**FIGURE 5:**
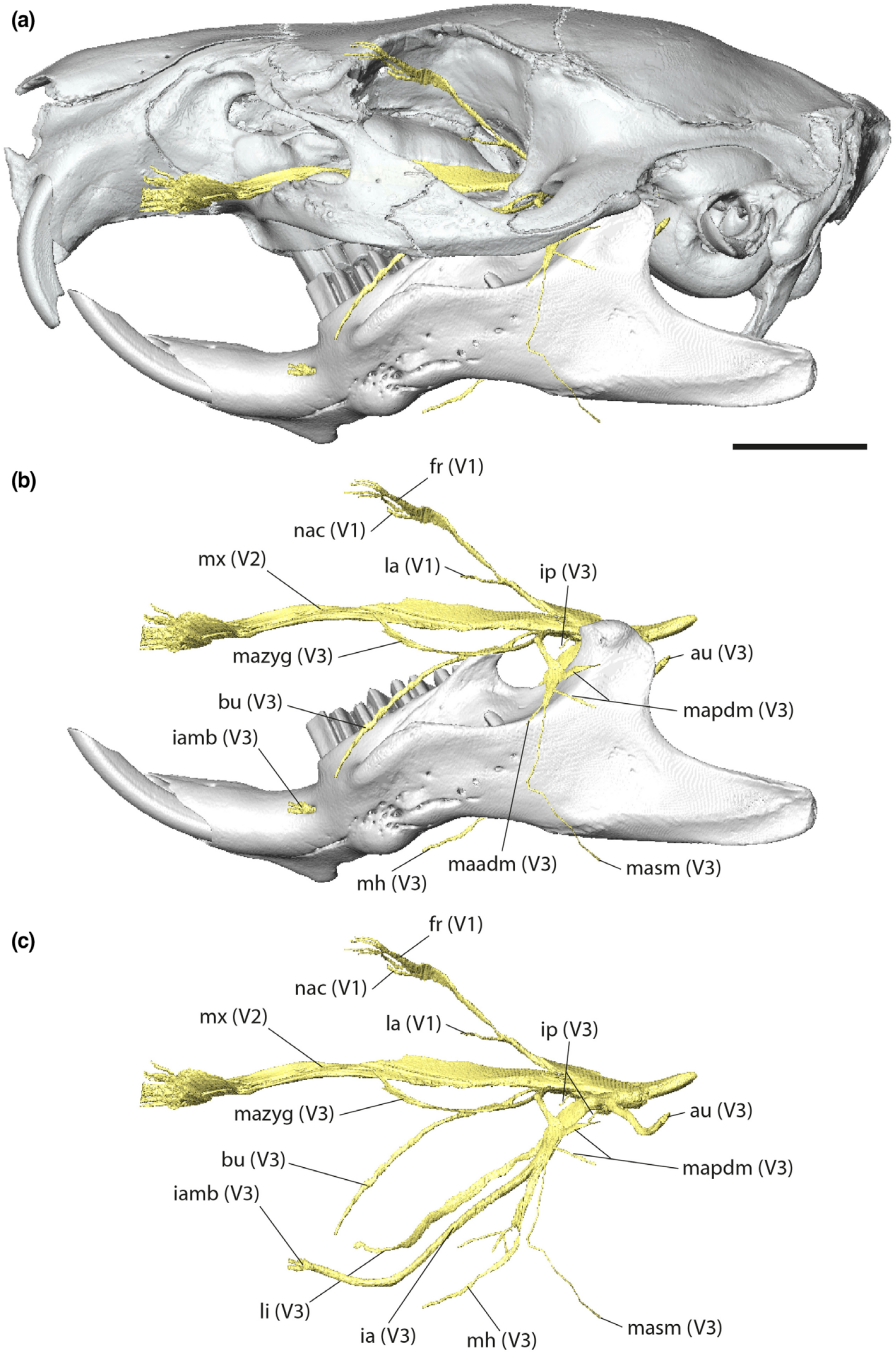
Virtual dissection of the trigeminal nerve of *Cavia porcellus* in lateral view: (a), with the cranium and the mandible; (b), with the mandible only; (c), without the cranium and the mandible. *au*, auriculotemporal nerve; *bu*, buccinator nerve; *fr*, frontal nerve; *iamb*, mental branch of inferior alveolar nerve; *ia*, inferior alveolar nerve; *ip*, internal pterygoid nerve; *la*, lacrimal nerve; *li*, lingual nerve; *ma*, masseteric nerve; *maadm*, branch of the masseteric nerve for the anterior deep masseter muscle; *mapdm*, branches of the masseteric nerve for the posterior deep masseter muscle; *masm*, branch of the masseteric nerve for the superficial masseter muscle; *mazyg*, branch of the masseteric nerve for the zygomaticomandibular muscle; *mh*, mylohyoid nerve; *mx*, maxillary nerve; *nac*, nasociliary nerve; V1, ophthalmic division; V2, maxillary division; V3, mandibular division. Scale bars are 10 mm.

In the following comparative description, each of the three main branches of the trigeminal nerve (i.e., the ophthalmic, maxillary, and mandibular divisions) is considered individually. They are first described in detail in *Rattus norvegicus* (Figures 3 and S2) and then compared to those of *Sciurus vulgaris* (Figures 4 and S2) and *Cavia porcellus* (Figures 5 and S2). Although several specimens of *Sciurus* and *Cavia* were dissected using conventional dissection techniques, no major interindividual differences in nerve conformation were observed among the branches that could be identified. These observations pertained primarily to the main nerve branches, as the more distal terminal branches were too small to be reliably observed or characterized.

### 3.1 Ophthalmic nerve (V1)

The ophthalmic nerve, the first division of the trigeminal nerve (V1), divides into three branches: the lacrimal (la), frontal (fr), and nasociliary (nac) (Greene, 1935). The lacrimal branch innervates the intraorbital lacrimal gland and the conjunctiva. The frontal branch traverses the orbit medially to the eye, exits in the region of the upper eyelid, and sends branches to this area before innervating the forehead skin. The nasociliary branch starts ventrally to its division with the frontal branch, after which it enters the cranial cavity via the ethmoid foramen. It then continues anteriorly until it reaches the cribriform plate, which it pierces in order to enter the nasal cavity and reach the nasal mucosa. The nasociliary branch also innervates the skin surrounding the nose between the nasal bone and the nasal cartilage after exiting the nasal cavity (Greene, 1935).

**FIGURE 6:**
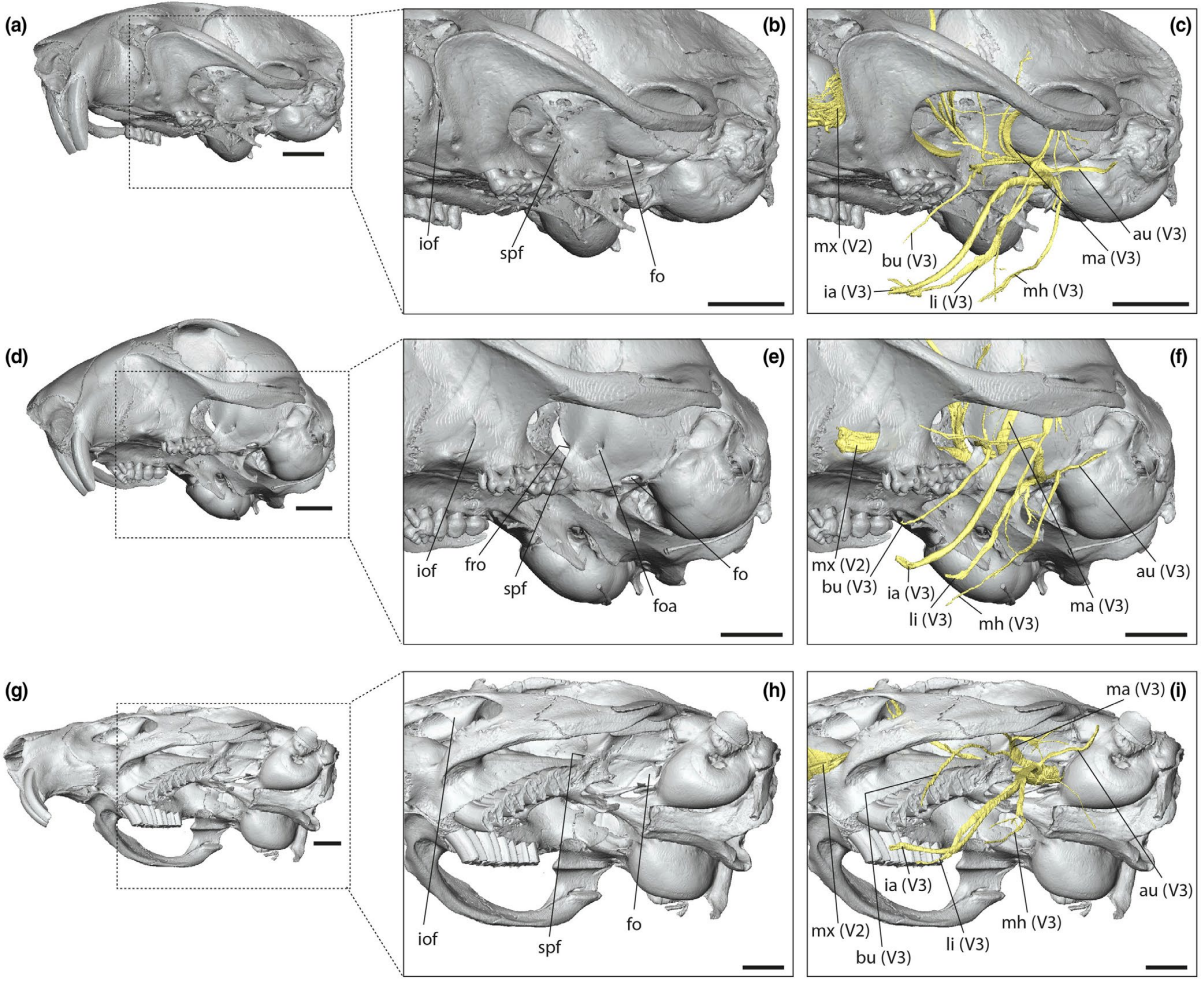
Trigeminal nerve and its relationship with cranial foramina in *Rattus norvegicus* (a, b, and c), *Sciurus vulgaris* (d, e, and f), and *Cavia porcellus* (g, h, i). (a, d, and g) Anteroventral view of the skull. (b, e, and h) Zoom on the posterior part of the skull with the trigeminal nerve removed. (c, f, and i) Zoom on the posterior part of the skull with the trigeminal nerve. *au*, auriculotemporal nerve; *bu*, buccinator nerve; *fo*, foramen ovale; *foa*, accessory foramen ovale; *fro*, foramen rotundum; *ia*, inferior alveolar nerve; *iof*, infraorbital foramen; *li*, lingual nerve; *ma*, masseteric nerve; *mh*, mylohyoid nerve; *mx*, maxillary nerve; spf, sphenorbital fissure; V2, maxillary division; V3, mandibular division. Scale bars are 5 mm.

In *Rattus* (Figures 3 and S2), the ophthalmic nerve diverges from the maxillary nerve well posteriorly to the opening of the sphenorbital fissure and then passes through the fissure, which is indistinguishable from the foramen rotundum (Figure 6). The lacrimal, frontal, and nasociliary branches diverge from the main branch anterior to the sphenorbital fissure. These branches are never in contact with the mandible. Posteriorly, the frontal and lacrimal branches follow the orbital wall and the anteriormost fibers of the medial temporal muscle, without innervating them, close to the medial border of the coronoid process. The nasociliary branch is the longest branch of the ophthalmic nerve, but it does not contact any of the masticatory muscles. Its main branch crosses the medial wall through the anterior ethmoidal foramen, anteriorly to the other branches of the ophthalmic nerve; it then turns dorsally toward the nasal cavity to reach the level of the dorsalmost part of the frontal nerve, close to the cranial roof. The smaller secondary nasociliary branch remains within the orbit, and follows the orbital wall anterodorsally until it almost reaches the cranial roof.

In *Sciurus* (Figures 4 and S2), the configuration is quite similar, although the foramen rotundum and the sphenorbital fissure are distinct but fused (Figure 6), with the ophthalmic nerve being restricted to the foramen rotundum. The lacrimal branch follows the orbital wall and the anterior fibers of the medial temporal muscle posteriorly. The frontal branch is the thickest. It projects anterodorsally and, compared to the lacrimal branch, terminates very dorsally. It follows the path of the nasociliary branch ventrally and exits the skull dorsally through a supraorbital foramen located midway along the dorsal margin of the orbit. The nasociliary branch has no contact with the masticatory muscles; it follows the path of the frontal branch ventrally and diverges from it at mid‐height of the orbit to cross the medial wall through the anterior ethmoidal foramen.

**FIGURE 7:**
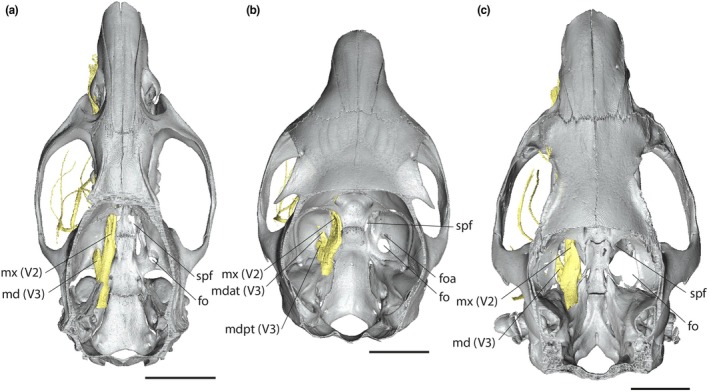
Dorsal view of the endocranial path of the trigeminal nerve and its relationship with cranial foramina in *Rattus norvegicus* (a), *Sciurus vulgaris* (b), and *Cavia porcellus* (c) (cranial roof virtually removed). Note the differences between the foramen ovale and its association to an accessory foramen ovale in *Sciurus*. In *Sciurus*, the mandibular nerve divides into anterior and posterior trunks (*mdat* and *mdpt*) within the cranial cavity, whereas in *Rattus* and *Cavia*, this bifurcation occurs outside of it. *fo*, foramen ovale; *foa*, accessory foramen ovale; *md*, mandibular nerve; *mdat*, anterior trunk of the mandibular nerve; *mdpt*, posterior trunk of the mandibular nerve; *mx*, maxillary nerve; *spf*, sphenorbital fissure; V2, maxillary division; V3, mandibular division. Scale bars are 10 mm.

In *Cavia* (Figures 5 and S2), the innervation pattern of the ophthalmic nerve significantly differs from that of *Rattus* and *Sciurus*. As in *Rattus*, the foramen rotundum and the sphenorbital fissure cannot be distinguished (Figures 6 and 7). The ophthalmic branch has no contact with the masticatory musculature; it is positioned medially compared to the temporal muscle. The most dorsal part of the origin of the internal pterygoid muscle, high in the orbit, passes close medially to the ophthalmic branch at the level of the bifurcation between the nasociliary/frontal branches and the lacrimal branch. The lacrimal branch is short and straight with an anteroposterior orientation. It diverges approximately halfway between the nasociliary/frontal branch bifurcation and the ophthalmic/maxillary nerve main bifurcation. The frontal branch is wide and flattened dorsally; it crosses the orbit medially and is multifurcating dorsal to the anterior fronto‐orbital ridge. The nasociliary branch is short, subdivided, and diverges dorsally from the frontal branch close to the dorsal border of the orbit.

### 3.2 Maxillary nerve (V2)

The maxillary nerve, the second division of the trigeminal nerve, is composed of numerous branches that supply different parts of the head from anterior to posterior: the side of the nose and the skin of the muzzle, the nasal and oral mucosae, the skin and mucosa of the cheeks, the skin of the lower and upper lips, the gums, the molars and incisors, the lower eyelid, and the dura mater membrane (Greene, 1935). Many of the smallest accessory branches of the maxillary nerve were not visible on the CT images and could not be reconstructed, especially the middle meningeal, zygomaticotemporal, zygomaticofacial, superior alveolar, inferior palpebral, external nasal, and superior labial branches.

In *Rattus* (Figures 3 and S2), the maxillary nerve exits the skull with the ophthalmic division (V1) through the sphenorbital fissure and crosses the orbit via a deep groove (the pterygopalatine fossa *sensu* Greene, 1935) at the ventro‐posterior border of the temporal fossa. The nerve then enters the orbit, crosses the maxillary foramen and the infraorbital canal, and exits through the infraorbital foramen (anterior end of the canal on the rostrum). The maxillary nerve never contacts the mandible or any of the masticatory muscles, not even the infraorbital part of the zygomaticomandibular muscle (ioZM, Figures 2b and 9b), to which it remains very close in the infraorbital foramen, but is always ventrally confined to the ventral notch of the infraorbital canal defined by the zygomatic plate.

In *Sciurus* (Figures 4 and S2), the innervation pattern is essentially the same as in *Rattus*, except that no part of the zygomaticomandibular muscle passes through the infraorbital foramen, leaving only the maxillary nerve to cross this foramen. The nerve diverges laterally halfway through the tooth row above the M1 as it enters the infraorbital canal.

In *Cavia* (Figures 5 and S2), the main branch of the maxillary nerve follows a similar course as in *Rattus* and *Sciurus*. The infraorbital part of the zygomaticomandibular muscle is large and crosses the infraorbital foramen (Figure 9f), but is always separated medially from the maxillary nerve by a small bony wall that wraps the nerve laterally.

### 3.3 Mandibular nerve (V3)

**FIGURE 8:**
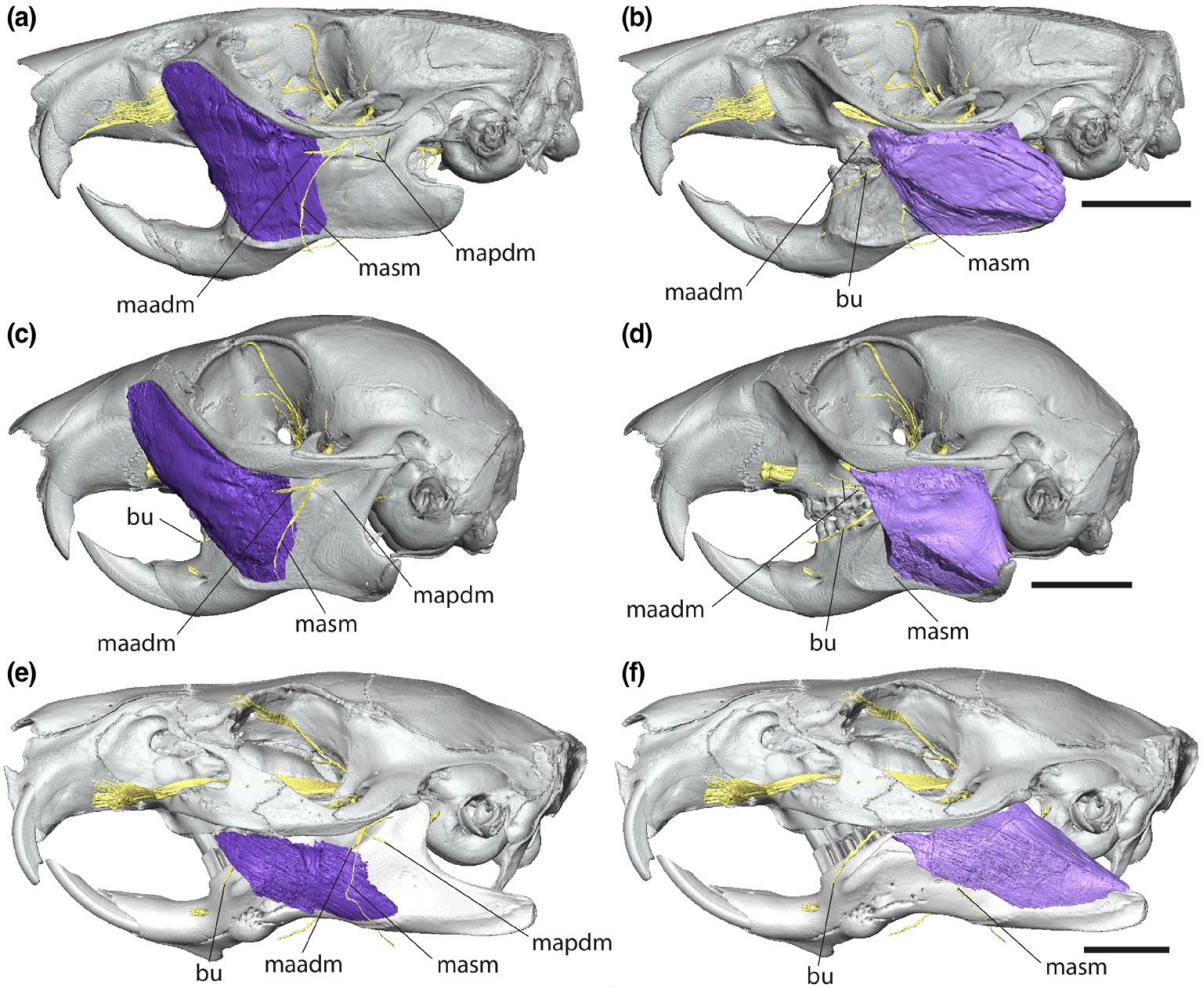
Left lateral view of the 3D reconstruction of the skull and anterior (purple, left) and posterior (lilac, right) deep masseter muscle in *Rattus norvegicus* (a, b), *Sciurus vulgaris* (c, d), and *Cavia porcellus* (e, f). The superficial masseter was removed because it partly covers the deep masseter muscle and all the associated branches of the trigeminal nerve. The labels are for the branches of the trigeminal nerve (in pale yellow). *bu*, buccinator nerve; *maadm*, branch of the masseteric nerve for the anterior deep masseter muscle; *mapdm*, branches of the masseteric nerve for the posterior deep masseter muscle; *masm*, branch of the masseteric nerve for the superficial masseter muscle. Scale bars are 10 mm.

**FIGURE 9:**
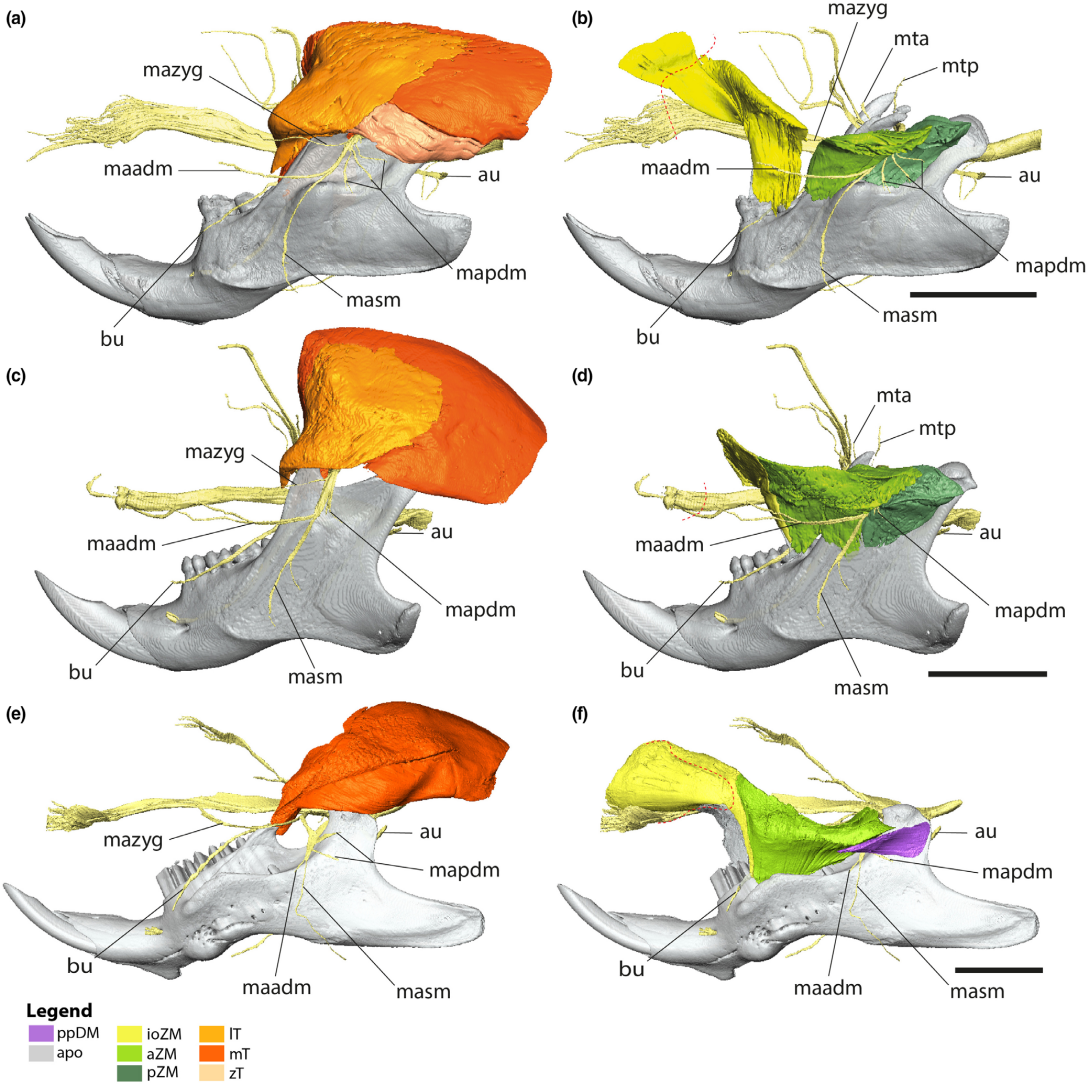
Left lateral view of the 3D reconstruction of the mandible, temporal (left), and zygomaticomandibular (right) muscles in *Rattus norvegicus* (a, b), *Sciurus vulgaris* (c, d), and *Cavia porcellus* (e, f). The labels are for the branches of the trigeminal nerve (in pale yellow). The color code is for all muscle parts. *au*, auriculotemporal nerve; *bu*, buccinator nerve; *mazyg*, branch of the masseteric nerve for the zygomaticomandibular muscle; *mta*, medial temporal anterior nerve; *mtp*, medial temporal posterior nerve; *maadm*, branch of the masseteric nerve for the anterior deep masseter muscle; *mapdm*, branches of the masseteric nerve for the posterior deep masseter muscle; *masm*, branch of the masseteric nerve for the superficial masseter muscle. The muscle abbreviations are the same as in Figure 1; ppDM is only present in *Cavia*. Scale bars are 10 mm.

**FIGURE 10:**
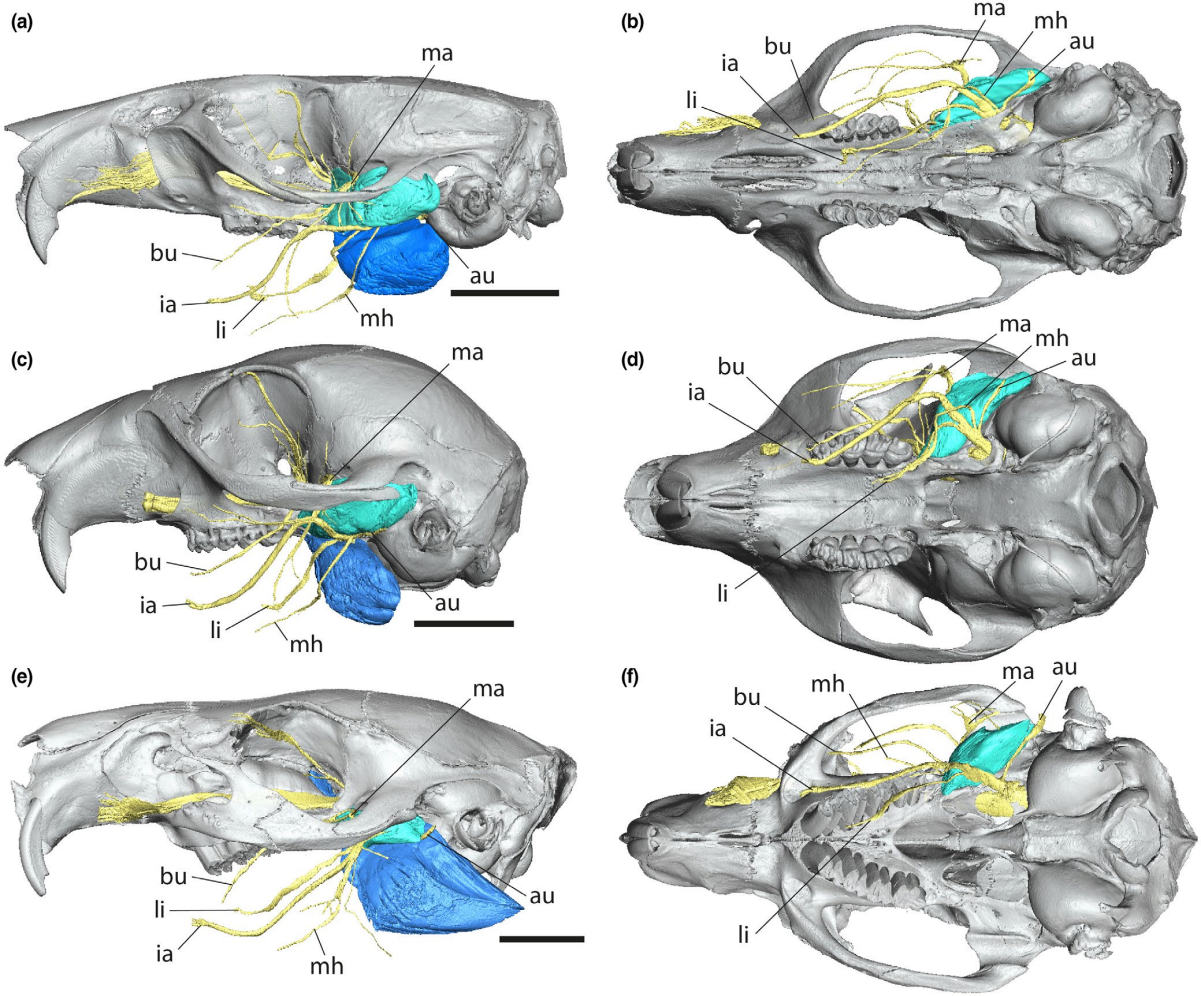
Left lateral (left) and ventral (right) views of the 3D reconstruction of the cranium, and both internal (light blue) and external (blue) pterygoid muscles in *Rattus norvegicus* (a, b), *Sciurus vulgaris* (c, d), and *Cavia porcellus* (e, f). The labels are for the branches of the trigeminal nerve (in pale yellow). *au*, auriculotemporal nerve; *bu*, buccinator nerve; ia, inferior alveolar nerve; *li*, lingual nerve; *ma*, masseteric nerve; *mh*, mylohyoid nerve. Scale bars are 10 mm.

The mandibular nerve, the third division of the trigeminal nerve, has sensory and motor roots (Greene, 1935). It is the most complex of the three trigeminal divisions. Its numerous branches innervate various regions of the head, from anterior to posterior: the skin of the chin and the mucosa of the lower lip, the transverse mandibular muscle, the anterior belly of digastric muscle, the tongue, the sublingual glands, the mucosa of the mouth, the gums, the lower cheek teeth and incisors, the buccinators, the mylohyoid muscle, the masticatory muscles (i.e., masseter, zygomaticomandibular, temporal, and pterygoid muscles; Figures 8, 9, 10), the skin of the temporal region, the pinna of the ear, the external acoustic meatus, the temporomandibular joint, the parotid glands, and the dura mater (Greene, 1935). As for the maxillary division, some of the smallest accessory branches of the mandibular nerve could not be reconstructed due to insufficient resolution of the CT images, especially the inferior dental, incisor, anterior auricular, articular, parotid, superficial temporal branches, and the nervus spinosus.

In *Rattus* (Figure 6), all the branches but the internal pterygoid nerve (ip) go through the foramen ovale. Upon exiting the skull through the foramen ovale, the mandibular nerve gives rise to the nervus spinosus and internal pterygoid nerve. The nervus spinosus is supposed to reenter the skull (Greene, 1935), but was not observed here. The internal pterygoid nerve goes through the window opened by the alisphenoid canal on the pterygoid wing. Then, the mandibular nerve further divides into two main complexes: the anterior trunk subdivides into the masseteric (ma), medial temporal (mt, deep temporal *sensu* Greene, 1935), buccinator (bu; long buccal), and external pterygoid (ep) branches; while the posterior trunk subdivides into the auriculotemporal (au), lingual (li), and inferior alveolar (ia) nerves (Figure 3).

In *Sciurus* (Figures 4, 6e,f, 7b), the mandibular nerve passes through the foramen ovale and the accessory foramen ovale (*foramen ovale accessorius*, *sensu* Bertrand et al., 2018). The latter corresponds to two confluent foramina, the most anteroventral for the buccinator nerve and the more posterodorsal for the masseteric nerve. All the other branches go through the foramen ovale, including the internal pterygoid nerve that has no interaction with the pterygoid wing, unlike in *Rattus*.

In *Cavia* (Figures 5, 6g–i, and 7c), the mandibular nerve passes through the very enlarged foramen ovale, from which the maxillary branch is also visible in ventral view.

#### 3.3.1 Anterior trunk

##### Buccinator nerve

In *Rattus* (Figures 3 and S2), the buccinator nerve passes between the root of the coronoid process (medially) and the tooth row (laterally) to reach the buccinator muscle at the level of the anterior part of the tooth row. It passes anteroventrally to the temporal muscle and medially to the zygomaticomandibular muscle (Figure 9a). It crosses the external pterygoid muscle laterally (Figure 10a). Close to the root of the buccal nerve, a small branch, the anterior medial temporal nerve, diverges dorsally to supply the medial temporal muscle (Figure 9). Both the anterior medial temporal nerve and the buccal nerve itself cross the external pterygoid muscle, with the buccal nerve crossing it more anteriorly (Figure 10a). Otherwise, the buccal nerve follows the most medial fibers of the external pterygoid muscle (ePT) medially.

In *Sciurus* (Figures 4 and S2), the buccinator nerve follows the same path as in *Rattus*, except that it does not cross the external pterygoid muscle, remaining instead dorsomedial to it (Figure 10c,d). The nerve passes anteroventrally to the medial border of the medial temporal muscle and medially to the zygomaticomandibular muscle (Figure 9c,d). The anterior medial temporal nerve is also present and diverts dorsally to supply the medial temporal muscle. Like the buccinator nerve, this nerve does not cross the external pterygoid and remains dorsal to it.

In *Cavia* (Figures 5 and S2), the buccinator nerve passes between the tip of the coronoid process medially and the tooth row laterally to reach the buccinators at the level of the anterior part of the tooth row, medially to the zygomaticomandibular muscle (Figure 9e,f).

##### Masseteric nerve

After bifurcating from the rest of the anterior trunk, the masseter nerve in *Rattus* wraps the external pterygoid muscle dorsally (Figure 10a,b), but passes ventrally to the insertion of the medial temporal muscle. The external pterygoid muscle is enclosed between the masseteric nerve dorsally and the posterior trunk ventrally. It then passes through the mandibular notch (i.e., the sigmoid notch defined as the gap between the coronoid process and the condyle; Figure 3) and the zygomaticomandibular complex to reach the masseter complex (Figures 8a,b, 9b). A branch (*mazyg*, Figure 9a,b) diverges early from the main branch as it passes laterally through the mandibular notch. The *mazyg* branch crosses the anterior part of the zygomaticomandibular muscle longitudinally in its distal portion; it then crosses the anterior part medially and closely follows its medial border as well as that of the infraorbital part of the zygomaticomandibular muscle to innervate the latter near the junction between the two parts of the muscle (rostral vs zygomatic). The *mazyg* likely innervates the anterior part of the zygomaticomandibular muscle along its length, but none of the terminal branches are visible on the CT images. On the other hand, the main branch of the masseteric nerve continues ventrally to the most posterior fibers of the anterior part of the zygomaticomandibular muscle, but dorsally to the most anterior fibers of the posterior part of the zygomaticomandibular muscle (pZM, Figure 9b). All the most distal masseteric branches diverging from this main branch closely follow the lateral border of the mandible to supply successively different muscle parts (Figure 8a,b). Three branches (*mapdm*, Figure 8a) supply the posterior deep masseter, one main branch (*maadm*, Figure 8a,b) supplies the anterior deep masseter, and one main branch (*masm*, Figure 8a,b) supplies the superficial masseter. The posterior part of the zygomaticomandibular muscle is medial to the branches innervating the posterior deep masseter, while the anterior part of the zygomaticomandibular muscle is medial and dorsal to the branch innervating the anterior deep masseter (*maadm*).

The pattern of innervation of the masseteric nerve in *Sciurus* (Figure 4) is essentially the same as in *Rattus*. The early bifurcating branch (*mazyg*) supplying the zygomasseteric complex (Figure 9c) crosses the anterior part of the zygomaticomandibular muscle along its entire length, but its course cannot be followed until it reaches the infraorbital part of the zygomaticomandibular muscle. Two branches (*mapdm*, Figure 8c) of the masseteric nerve supply the posterior deep masseter, two branches (*maadm*, Figure 8c,d) separated along their entire length supply the anterior deep masseter, one main branch (*masm*) plus an accessory branch supply the superficial masseter muscle (Figure 8c,d).

In *Cavia*, the pattern of innervation of the masseteric nerve differs from that of *Rattus* and *Sciurus* (Figures 5 and S2). The early bifurcating branch supplying the zygomasseteric complex (*mazyg*, Figure 9f) is thick, much thicker than the other masseteric branches. Then, it crosses the anterior part of the zygomaticomandibular muscle and follows its medial border along its entire length to innervate the infraorbital part of the zygomaticomandibular muscle (Figure 2e,f). The posterior end of the anterior part of the zygomaticomandibular muscle is enclosed by the bifurcation defined by the *mazyg* and the main masseteric branch (Figure 9f). The main branch of the nerve lies ventrally to the most posterior fibers of the anterior part of the zygomaticomandibular muscle, and medially to the posterior part of the deep masseter. All parts of the deep and the superficial masseters are supplied individually by a single branch diverging successively from the main branch (Figure 8e,f). The branch innervating the superficial masseter (*masm*) is long, very thin, and ventrally oriented (Figures 5 and 8e). The external pterygoid muscle is crossed dorsally by the anterior trunk and then enclosed between the buccal and masseteric nerves (dorsally), and the posterior trunk (laterally) (Figure 10).

##### Posterior medial temporal nerve

In *Rattus*, this nerve (*mtp*, medial temporal posterior nerve, Figure 3) diverges from the main masseteric branch. After the split between the main division separating the anterior and posterior trunks, it quickly turns dorsally, adopting an almost dorsoventral orientation, in order to reach the medial temporal muscle (Figure 9a,b).

The course of the posterior medial temporal nerve in *Sciurus* resembles the condition observed in *Rattus* (Figure 9a,b), but it was not observed in *Cavia* (Figure 9e,f).

##### External pterygoid nerve

In *Rattus*, the external pterygoid nerve enters the external pterygoid muscle dorsally, but ventrally to the anterior trunk (Figures 3 and 10a,b). It is located between the anterior and the posterior medial temporal nerves, and diverges just posteriorly to the division between the posterior medial temporal nerve and the masseteric nerve.

The external pterygoid nerve could not be observed in *Sciurus* (Figures 4 and S2).

In *Cavia*, a tiny branch of the external pterygoid nerve is visible ventrally to the anterior trunk, posteriorly to the division between the masseteric and the buccal branches, but anterior to the main division between the anterior and posterior trunks.

#### 3.3.2 Posterior trunk

**FIGURE 11:**
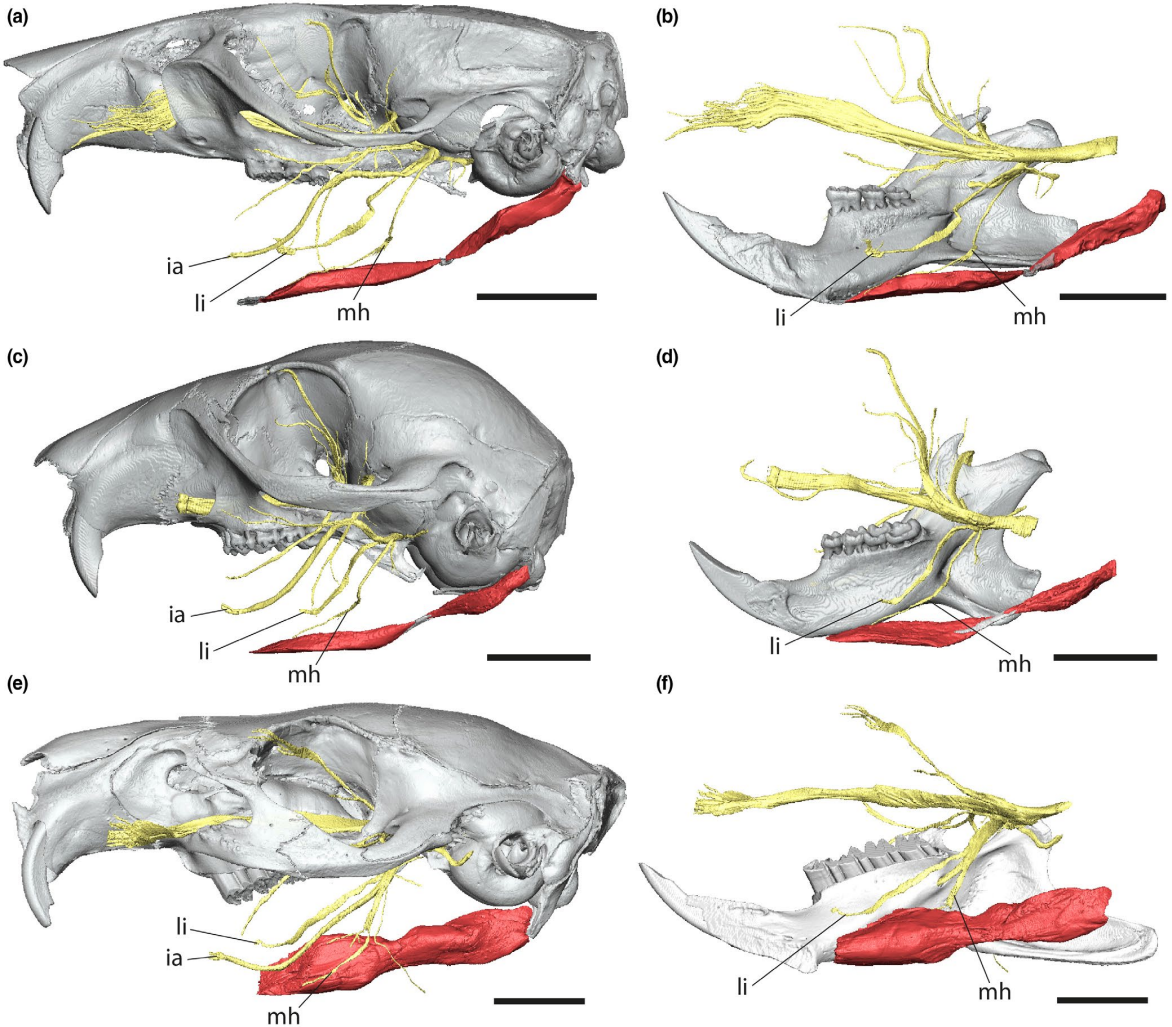
Left lateral view of the 3D reconstruction of the cranium (left), mandible (right), and digastric muscle in *Rattus norvegicus* (a, b), *Sciurus vulgaris* (c, d), and *Cavia porcellus* (e, f). The labels are for the branches of the trigeminal nerve (in pale yellow). *iamb*, mental branch of inferior alveolar nerve; *li*, lingual nerve; *mh*, mylohyoid nerve. Scale bars are 10 mm.

In *Rattus*, the posterior trunk of the mandibular nerve divides into four main branches, from posterior to anterior: the auriculotemporal (au), the mylohyoid (mh), the inferior alveolar (ia), and the lingual (li) nerves (Figures 3 and S2). The posterior trunk surrounds the internal pterygoid dorsally, especially the lingual and mylohyoid nerves (Figure 10a,b). All branches of the posterior trunk pass between the two pterygoid muscles. Immediately after diverging from the posterior trunk, the auriculotemporal nerve turns posteriorly, running along the medial side of the condylar process of the mandible to reach its lateral side just below the condyle at the level of the external auditory meatus (Figures 3, 9a,b, and 10a,b). The mylohyoid nerve is the most ventral branch of the posterior trunk; it branches off from the inferior alveolar nerve just before the latter enters the mandibular foramen (Figures 3, 6b,c, 9a,b, 10a,b, and 11). The mylohyoid nerve curves ventrally around the mylohyoid muscle, which it supplies, but also sends branches to the anterior belly of the digastric and transverse mandibular muscles anteriorly (Figure 11a,b). The inferior alveolar nerve follows the medial border of the mandibular ramus, then enters the mandibular foramen, and runs through the mandibular canal (Figure 3). Within the canal, small branches innervate the teeth, while the main branch continues its course within the canal to exit the mandible laterally at the mental foramen, anteriorly to the molars. The lingual nerve runs anteroventrally, entering the tongue and extending to its anterior tip.

The posterior trunk in *Sciurus* resembles that of *Rattus*, with a multifurcation of four main branches from posterior to anterior (Figures 4 and S2). In *Sciurus*, the inferior alveolar branch is much more posterodorsally located (Figures 4, 6e,f, and 10c,d), which explains the curved aspect of the branch itself and might be linked to the posterior development of the incisors.

In *Cavia* (Figures 5 and S2), the pattern of innervation of the posterior trunk is similar to that of *Rattus* and *Sciurus*. However, the large branch uniting the mylohyoid, inferior alveolar, and lingual nerves divide only in an anterior position at the level of the posterior end of the mandible (Figures 5, 6h,i, 10e,f, and 11e,f). The mylohyoid nerve bifurcates into four branches: three early branches are very close together and supply the mylohyoid muscle, while the most ventral is larger and very long, and supplies the digastric and transverse mandibular muscles anteriorly (Figures 5, 10e,f, and 11e,f).

## 4 DISCUSSION

### 4.1 Methodological advances and limits

Advances in technology are driving a great deal of research, particularly in the areas of imaging and functional simulations. Yet, despite the widespread use of imaging technology and shape analyses to study mineralized tissues (bones and teeth), soft tissues are far less frequently studied in rodents (e.g., Baverstock et al., 2013; Cox et al., 2020; Cox & Faulkes, 2014; Da Cunha et al., 2024; Ginot et al., 2018). This study offers a first three‐dimensional perspective on the anatomy of the fifth cranial nerve in rodents, significantly expanding the existing data for this mammalian group (Greene, 1935). We managed to successfully identify a diverse array of tissue types, from muscles and bones to nerves and tendons, across three representative rodent species. However, CT resolution remains a primary limiting factor as many finer nerve branches, likely either too small for contrast agents or beyond the resolution obtained, were simply not visible here. Notable examples include the smallest accessory branches of the maxillary and mandibular nerves in all species, or the posterior medial temporal nerve in *Cavia* and the external pterygoid nerve in *Sciurus*. Likewise, thin aponeuroses were not discernible. Reconstructing complex 3D anatomical structures presents significant challenges, with misidentification being a common pitfall due to the difficulty of fully interpreting intricate spatial relationships. While diceCT methods have been invaluable for characterizing the detailed muscular and nervous systems (e.g., Lessner et al., 2022; Lessner & Holliday, 2020), our findings show that certain aspects of the morphological complexity of the masticatory apparatus can still be easily overlooked or entirely missed. For small specimens such as rodents, using traditional dissection also has limitations because the fine anatomical details of nervous structure terminations are often only discernible in histologically prepared specimens (Hadlock et al., 2013; Maier et al., 2002; Maier & Schrenk, 1987). Deeply positioned or internal structures also pose a challenge during dissection, as their topological relationships are difficult to characterize effectively. In our study, this was particularly true for the pterygoid muscle and its conformation to the mandibular branch of the trigeminal nerve (Figure 10). As suggested in previous studies (Da Cunha et al., 2024; Lessner & Holliday, 2020; Melekian et al., 2025), we advocate for the combined use of traditional and 3D dissection techniques to fully capture anatomical variation, arguing that relying solely on diceCT methods risks generating new confusion rather than resolving existing controversies.

### 4.2 Consistency of the trigeminal nerve pathway across rodent species

The challenge of visualizing nerves in conventional dissections may account for the comparatively lower interest in the nervous system than in the musculoskeletal counterpart. However, this may also be attributed to the perceived high degree of intra‐ and interspecific variation, including asymmetry between the left and right sides at an individual level (e.g., Konschake et al., 2016; Sawyer et al., 2020; Toledo et al., 2023). Although fine‐scale terminal wiring may be more variable than other systems, a possibility that still needs to be tested, the core gross anatomy of cranial nerves seems to be highly conserved across tetrapod species (Edgeworth, 1935; Holliday & Witmer, 2007; Jones et al., 2019; Lessner et al., 2022; Lessner & Holliday, 2020; Rieppel, 1980; Säve‐Söderbergh, 1945; Zusi & Livezey, 2000), including at a specific or an individual level.

We demonstrate that the overall gross anatomy of the trigeminal nerve remains largely consistent across the three rodent species examined here. Our results highlight the potential for greater stability in nervous pathways than is generally recognized. The primary nerves (ophthalmic, maxillary, and mandibular divisions) exhibit very similar bifurcation patterns across species, as do some of the secondary branches, such as those of the anterior and posterior trunks of the mandibular nerve. Their spatial relationship with the skeletal and muscular system is also strongly conserved. Such a highly conserved gross anatomy of cranial nerves is likely crucial for their viability and function (Lundborg & Rydevik, 1973; Nieuwenhuys et al., 1998), and may also explain some skeletal differences. For example, a given nerve may bifurcate either within or outside the cranial cavity. When bifurcation occurs intracranially, each resulting branch requires a distinct foramen for passage. Such variation in bifurcation sites may account for the differences observed between the foramen ovale and the accessory foramen ovale in rats and squirrels (Figures 6 and 7). In rats, the mandibular nerve divides into anterior and posterior trunks outside the cranial cavity, whereas in squirrels, this bifurcation occurs within it, reflecting a link between nerve morphology and cranial skeletal modifications.

In general, the shape of the core nervous architecture was largely similar in squirrels and rats. In contrast, the nervous organization in Guinea pigs showed significant differences, characterized by a more compact morphology. This was mostly evident in the ophthalmic and masseteric nerves, which were flattened and typically had poorly differentiated terminal branches. To determine whether these observed differences are species‐specific or characterize broader taxonomic groups, potentially holding systematic significance, it is essential to expand the dataset and conduct further comparative analyses, similar to what has been proposed for arterial patterns in rodents (Bugge, 1985). Variation in the shape or position of foramina is often used in systematics (Carleton, 1980; Carleton & Musser, 1989; Hill, 1935; Musser & Newcomb, 1983; Wahlert, 1974, 1985), particularly for fossil forms. However, direct observations of the anatomy of the nervous tissue beyond model species are lacking, as are studies that systematically compare the branching patterns of cranial nerves. Moreover, the intraspecific variation of cranial foramina has rarely been examined in detail (e.g., Musser & Newcomb, 1983), while it might be substantial.

We also observed that the relative size of the main nerve branches remains consistent across the rodent species studied here, as in previously described mammal species (Evans & De Lahunta, 2012; Greene, 1935). The maxillary nerve is always larger than the mandibular and ophthalmic ones. Within the posterior trunk of the mandibular nerve, the inferior alveolar branch is consistently thicker than the lingual branch, which itself is thicker than the mylohyoid branch. A comparable pattern applies to the ophthalmic nerve, with the frontal branch being thicker than the nasociliary and lacrimal branches. In mammals, several studies have linked nerve size, or their associated osteological correlates, to ecological differences (Sanchez‐Villagra & Asher, 2002; Sulser & MacPhee, 2025). However, because the pattern observed here appears to be consistent across the three species examined, any attempt to relate these observations to their specific ecologies would remain highly tentative. Broader taxonomic sampling will therefore be necessary to investigate potential patterns of soft‐tissue convergence across additional rodent clades and species. In contrast, the relative size of the more peripheral branches could vary between species (see below) and might be related to the differential proportions of the associated innervated structure, or different behaviors associated with the innervated structures (e.g., Muchlinski et al., 2020).

Although the rodent cranial nervous system appears to exhibit a high degree of developmental canalization, it also demonstrates substantial plasticity at finer scales. This is particularly the case for the intricate pattern of terminal nerve branches, whose minute size makes them difficult or simply impossible to visualize and to follow with either conventional or digital dissection techniques. In most cases, the limited resolution of our dissection protocols, coupled with the small size of the specimens, precluded detailed characterization of fine‐scale terminal branching patterns and their relationship to internal muscle architecture. This contrasts with previous studies (e.g., Akita et al., 2019; Nakajima & Townsend, 2009; Shimokawa et al., 1998; Tomo et al., 1993) and reflects a limitation commonly observed in vertebrate studies employing similar techniques (Lessner et al., 2022; Lessner & Holliday, 2020). Such characterization would likely temper the perception of the high degree of conservation deduced from the gross anatomy of the cranial nerves.

### 4.3 The trigeminal nerve and its relationship to the masticatory musculature

Three main approaches have been used to classify muscles. These differ primarily based on the criteria used to define different muscle parts. The first approach, adopted by many early anatomists (e.g., Toldt, 1905; Tullberg, 1899; Wood, 1965), uses skeletal attachments to identify muscle parts. This can prove difficult due to the considerable disparity in mammalian cranial anatomy. The second approach relies on tendinous architecture (e.g., Gambaryan et al., 1980; Gaspard, 1954, 1971; Potapova, 2020; Yoshikawa & Suzuki, 1965). However, several studies have reported significant differences in the internal structure of the tendons of masticatory muscles between closely related species (Davis, 1964; Herring, 1980) and across a wide range of taxa (Druzinsky, 2010; Gaspard, 1954, 1971; Yoshikawa & Suzuki, 1965). The effectiveness of this approach is also influenced by the mode of preservation of the specimens. While these aponeuroses are typically well defined in fresh specimens, we observed that they can be difficult to delineate in specimens preserved in alcohol and particularly challenging to identify when examined using diceCT techniques. A third approach defines muscles based on their internal architecture and innervation (e.g., Akita et al., 2019; Nakajima & Townsend, 2009; Shimokawa et al., 1998; Tomo et al., 1993). However, Koppe et al. (1988) demonstrated in pigs that the nervous architecture does not always correspond to the internal organization of the masseter muscle. Unlike other mammalian groups, the innervation pattern of rodent masticatory muscles remains largely unexplored, except for the rat (Greene, 1935). The masseteric branch is the only exception, as its position relative to the parts of the masseter muscle has often been described (Boller, 1970; Druzinsky, 2010; Druzinsky et al., 2011; Fabre et al., 2017; Gambaryan et al., 1980; Hautier & Saksiri, 2009; Hill, 1937; Kesner, 1980; Satoh & Iwaku, 2004, 2006; Tullberg, 1899; Voss, 1988; Woods, 1972; Woods & Howland, 1979) and used to define and name some muscular parts (e.g., posterior deep masseter and posterior zygomaticomandibular muscles).

Some developmental studies indicate that muscle compartmentalization is closely associated with innervation, with motor unit territories confined to distinct neuromuscular compartments (Herring et al., 1991; Langenbach & Weijs, 1990; Weijs et al., 1993). Studies have then proposed that the intermuscular pathways of the trigeminal nerve reliably partition muscles into groups considered to be homologous (Edgeworth, 1935; Holliday & Witmer, 2007; Säve‐Söderbergh, 1945; Zusi & Livezey, 2000). In mouse craniofacial development, first‐arch mesoderm begins expressing myogenic regulatory factors such as MyoD and Myf5 around E10–10.5, marking the onset of muscle precursor specification (Yahya et al., 2022). Proper patterning and differentiation of these precursors require cranial–neural–crest‐derived mesenchyme, as its ablation results in proliferative myoblasts that fail to differentiate, highlighting a critical role for the surrounding tissue (Rinon et al., 2007). Trigeminal nerve branches, including the mandibular branch, enter the first‐arch mesenchyme around E10.5 (Motahari et al., 2020). Although the extent of contact with myogenic precursors remains unclear, these projections position axons near developing muscles, providing the anatomical potential for interaction. In the end, differentiated muscle fibers, indicated by MyHC expression, appear first at E10.5 and become robust by E11.5 (Yahya et al., 2022).

We observed that this early developmental pattern is reflected in the positioning of the main branches of the trigeminal nerve relative to the main masticatory muscle complexes (i.e., masseter, zygomaticomandibular, temporal, and pterygoid muscles). Indeed, these main branches are often located between muscle complexes and rarely cross them, in contrast to more peripheral branches that cross the muscular masses to perform their motor function. This is particularly evident in the insertion area of the temporal muscle, which lies between the lingual branch (laterally) and both the masseteric and inferior alveolar branches (anterolaterally) in *Rattus* and *Sciurus*, and between the lingual branch (laterally) and the masseteric branches (anterolaterally) in *Cavia*. A similar arrangement is observed in the course of the posterior trunk of the mandibular nerve, which consistently passes between the internal and external pterygoid muscles (Figure 10).

The situation becomes more complex when examining the relationships between muscle subdivisions and their interactions with nerves, particularly in the masseter, which is the most differentiated masticatory muscle in rodents (Turnbull, 1970). The masseter muscle exhibits a nonhomogeneous architecture, characterized by internal tendon structures that partition it into discrete anatomical regions (e.g., Gambaryan et al., 1980; Potapova, 2020). These partitions are composed of neuromuscular compartments, consisting of motor units that can be activated independently or in coordinated groups and confer the ability to generate different forces (Widmer et al., 2007). Task‐specific activation patterns allow selective recruitment of individual compartments or their coordinated engagement with other masticatory muscles. Widmer et al. (2007) demonstrated that the development of masseter compartmentalization is largely preprogrammed before birth, with muscle–nerve interactions playing only a limited role in its anatomical patterning. The partitioning of this muscle into distinct compartments occurs concurrently with the branching of the trigeminal nerve in intact embryos. Using an embryonic mouse model lacking innervation, Widmer et al. (2007) found that although the overall architecture of the masseter forms normally, muscle volume is reduced, likely reflecting a decreased capacity to generate secondary myotubes in the absence of nerve input. These observations indicate that muscle–nerve interactions during development are essential for muscle maturation rather than for establishing the initial compartmentalization of the muscle. Although we showed that each compartment of the masseter is innervated by distinct nerve branches, this may explain the previously observed imperfect correspondence between its nervous architecture and internal organization (Koppe et al., 1988).

We propose that early multifurcation patterns, rather than finer terminal branching patterns, provide a more reliable criterion for distinguishing individual muscle subdivisions (Akita et al., 2019; Nakajima & Townsend, 2009; Shimokawa et al., 1998; Tomo et al., 1993). We showed that this approach is applicable in both traditional and virtual dissections, ensuring reproducible homology assessments across studies. This is particularly relevant when considering the masseteric nerve (Figure 2), which exhibits an early multifurcation into distinct branches that innervate the superficial masseter (*masm*), as well as the anterior (*maadm*) and posterior (*mapdm*) portions of the deep masseter (Figure 8). In *Rattus* and *Sciurus*, the course of the branch innervating the superficial masseter represents a particularly robust criterion for separating the anterior and posterior parts of the deep masseter (Fabre et al., 2017; Kesner, 1980; Voss, 1988). These muscle portions were traditionally further distinguished by differences in fiber orientation (e.g., Ball & Roth, 1995; Druzinsky, 2010; Fabre et al., 2017; Thorington & Darrow, 1996; Voss, 1988) and aponeurotic structures (Potapova, 2020). Consistent dissection outcomes across different rodent clades (i.e., mouse and squirrel‐related clades) were here achieved by first localizing the nerve and subsequently accounting for fiber orientation. In contrast, in *Cavia*, the branch innervating the superficial masseter is relatively thin, and the distinction between the anterior and posterior portions of the deep masseter is poorly defined in both traditional and virtual dissections. The branch that supplies the anterior deep masseter is also underdeveloped compared to that of *Rattus* and *Sciurus*. Taken together, these observations suggest reduced anteroposterior compartmentalization of the deep masseter in this taxon, which may explain why it was not recognized in previous studies (Álvarez et al., 2023; Cox & Jeffery, 2011; Woods, 1972) and why delineating these parts proved difficult in both conventional and virtual dissections.

The masseteric nerve also typically crosses the zygomaticomandibular muscle masses, allowing the posterior parts in *Rattus* and *Sciurus* (Figure 9) to be identified, which appears to serve a function distinct from that of the rest of the zygomaticomandibular complex (Satoh & Iwaku, 2004, 2006). This region is variably observed in related species (Boller, 1970; Druzinsky, 2010; Gambaryan et al., 1980; Hautier & Saksiri, 2009; Hill, 1935; Satoh & Iwaku, 2004, 2006; Tullberg, 1899; Woods, 1972; Woods & Howland, 1979) and is absent in anomaluromorphs (Da Cunha et al., 2024) and *Cavia*. In the latter, it can be readily distinguished from the posterior part of the deep masseter (*sensu* Da Cunha et al., 2024), which displays distinct origin and insertion areas and is innervated by the masseteric nerve (*mapdm*, Figure 9). In contrast, the zygomaticomandibular muscle is supplied by a distinct branch (*mazyg*, Figure 9e,f) that diverges from the masseteric nerve just before its characteristic multifurcation for the superficial and deep masseters. The fact that it is innervated by the masseteric branch has led to suggestions that the zygomaticomandibular muscle should be considered part of the masseteric complex in the past (Druzinsky et al., 2011; Shimokawa et al., 1999, 2002; Yoshikawa & Suzuki, 1965). In *Sciurus*, the *mazyg* branch crosses the full length of the zygomaticomandibular muscle and is not visible in the dorsal view. In *Rattus* and *Cavia*, which exhibit a highly developed infraorbital part of the zygomaticomandibular muscle, the branch runs medially along the muscle, likely providing innervation along its path and typically terminating within the infraorbital portion. The pronounced medial development of the infraorbital part of zygomaticomandibular muscle within the infraorbital region may explain the more medial course of this nerve in these taxa. The anterior termination of this branch may also serve as an additional criterion for distinguishing the infraorbital part of zygomaticomandibular muscle from the anterior part of zygomaticomandibular muscle together with differences in muscle origin, fiber orientation and the presence of tendinous structures (Fabre et al., 2017; Gambaryan et al., 1980; Hautier & Saksiri, 2009; Potapova, 2020; Satoh & Iwaku, 2004, 2006; Voss, 1988; Woods, 1972; Woods & Howland, 1979). In contrast, we observed no association between innervation patterns and compartmentalization in the temporal muscle, with the two main temporal nerve branches (medial temporal anterior, *mta*; and medial temporal posterior, *mtp*; Figure 9) innervating only the medial portion of the temporal muscle.

## 5 CONCLUSION

Our findings demonstrate that the gross anatomy of the trigeminal nerve remains largely unchanged across the three rodent species examined. However, although the rodent nervous system seems to exhibit a high degree of developmental canalization, it also demonstrates notable plasticity at finer anatomical scales. Some observed variations, such as apparently missing nerve branches, were likely attributable to limitations in resolution rather than true anatomical absence. We therefore strongly caution against drawing definitive conclusions from 3D data alone without first meticulously comparing it against physical dissections combined with detailed histological methods. We also demonstrated that the nervous pattern in Guinea pigs differs markedly from that in squirrels and rats, and that these differences are associated with modifications in skeletal structures and muscles. Further comparative studies are necessary to assess whether some of these differences have systematic significance. This issue is particularly relevant in paleontological contexts, where innervation patterns are usually inferred from extant model organisms or domestic animals (e.g., de Muizon et al., 2015; Wahlert, 1974; Wible & Shelley, 2020). The classification of muscles can vary substantially depending on the criteria employed, such as muscle attachments, tendinous architecture, or patterns of innervation. These discrepancies highlight the inherent challenges and variability in defining discrete muscular parts and suggest that no single method is entirely satisfactory. We advocate for a holistic and descriptive approach that incorporates multiple criteria, with the aim of improving the reproducibility and consistency of muscle compartmentalization across studies and between groups. The evolution of the trigeminal nerve in mammals probably reflects adaptations to different ecologies and feeding strategies. Although the present sample is too limited to support robust ecological interpretation, broader comparative analyses could help clarify the relationship between modifications of the rodent masticatory apparatus and their specific dietary and sensory specializations.

## Supporting information


**Figure S1.** The arrangement of the trigeminal nerve (outlined in yellow) as visualized from the diceCT in *Rattus norvegicus* (a, b), *Sciurus vulgaris* (c, d), and *Cavia porcellus* (e, f). Left, the dotted line indicates the position of the slides on the skull.

**Figure S2.** Virtual dissections of the trigeminal nerve of *Rattus norvegicus* (a, d, g), *Sciurus vulgaris* (b, e, h), and *Cavia porcellus* (c, f, i) in lateral view: with the cranium and the mandible (a–c); with the mandible only (d–f); without the cranium and the mandible (g, h, and i). *au*, auriculotemporal nerve; *bu*, buccinator nerve; *ep*, external pterygoid nerve; *fr*, frontal nerve; *iamb*, mental branch of inferior alveolar nerve; *ia*, inferior alveolar nerve; *ip*, internal pterygoid nerve; *la*, lacrimal nerve; *li*, lingual nerve; *ma*, masseteric nerve; *maadm*, branch of the masseteric nerve for the anterior deep masseter muscle; *mapdm*, branches of the masseteric nerve for the posterior deep masseter muscle; *masm*, branch of the masseteric nerve for the superficial masseter muscle; *mazyg*, branch of the masseteric nerve for the zygomaticomandibular muscle; *mh*, mylohyoid nerve; *mta*, medial temporal anterior nerve; *mtp*, medial temporal posterior nerve; *mx*, maxillary nerve; *nac*, nasociliary nerve; V1, ophthalmic division; V2, maxillary division; V3, mandibular division. Scale bars are 10 mm.

## Data Availability

The data that support the findings of this study are openly available in MorphoMuseuM at https://morphomuseum.com/.
