# Supplementary figures and images for: A 3D atlas of the trigeminal nerve and its relevance for comparative studies of the masticatory apparatus in rodents
Source: J Anat. 2026 Jul 13:10.1111/joa.70212. Online ahead of print. doi: 10.1111/joa.70212 (PMC13398523; doi:10.1111/joa.70212)

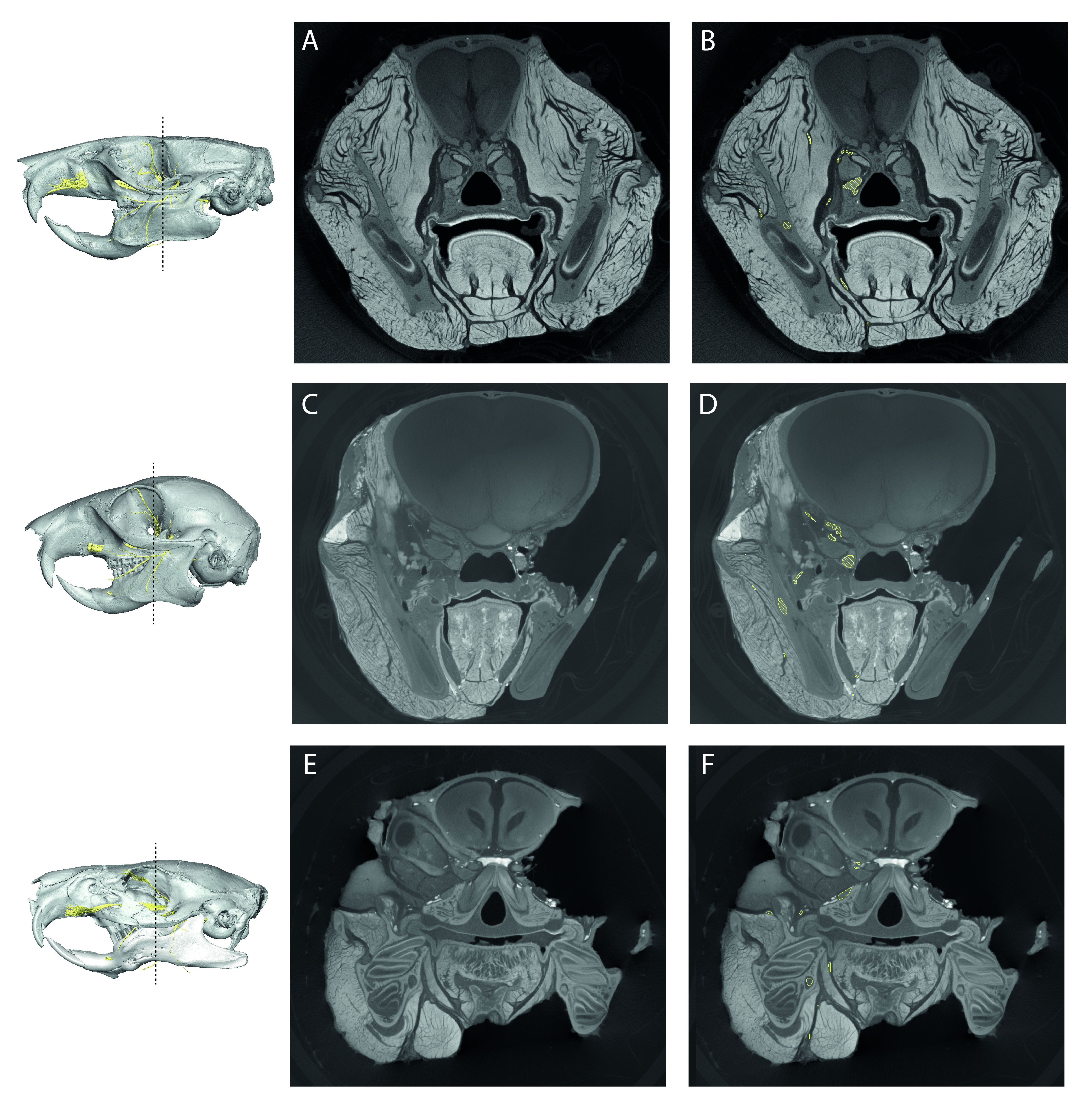

Supplement: Supplementary file 1 — Figure S1. The arrangement of the trigeminal nerve (outlined in yellow) as visualized from the diceCT in Rattus norvegicus (a, b), Sciurus vulgaris (c, d), and Cavia porcellus (e, f). Left, the dotted line indicates the position of the slides on the skull. [file JOA-9999-0-s001.jpg]

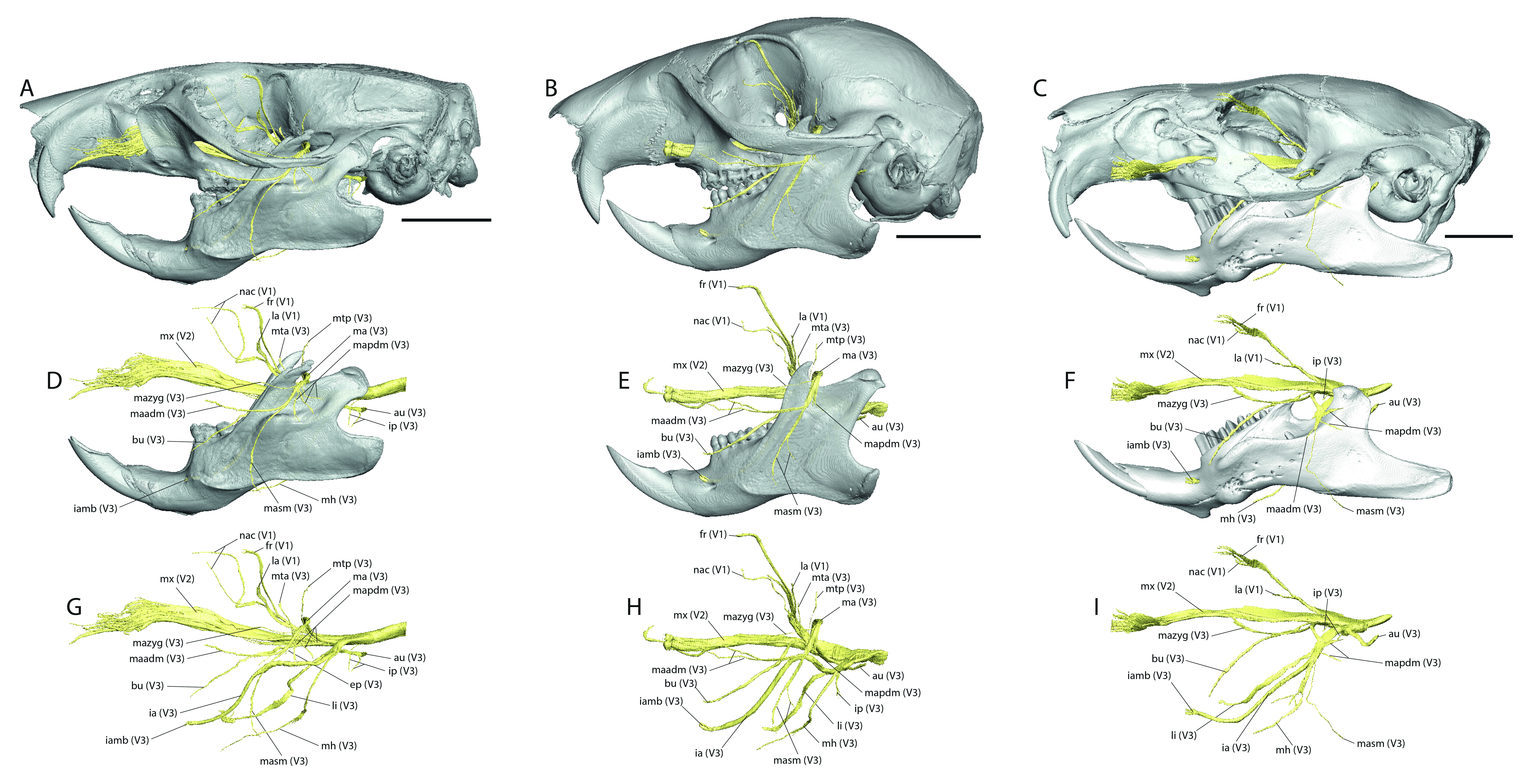

Supplement: Supplementary file 2 — Figure S2. Virtual dissections of the trigeminal nerve of Rattus norvegicus (a, d, g), Sciurus vulgaris (b, e, h), and Cavia porcellus (c, f, i) in lateral view: with the cranium and the mandible (a–c); with the mandible only (d–f); without the cranium and the mandible (g, h, and i). au, auriculotemporal nerve; bu, buccinator nerve; ep, external pterygoid nerve; fr, frontal nerve; iamb, mental branch of inferior alveolar nerve; ia, inferior alveolar nerve; ip, internal pterygoid nerve; la, lacrimal nerve; li, lingual nerve; ma, masseteric nerve; maadm, branch of the masseteric nerve for the anterior deep masseter muscle; mapdm, branches of the masseteric nerve for the posterior deep masseter muscle; masm, branch of the masseteric nerve for the superficial masseter muscle; mazyg, branch of the masseteric nerve for the zygomaticomandibular muscle; mh, mylohyoid nerve; mta, medial temporal anterior nerve; mtp, medial temporal posterior nerve; mx, maxillary nerve; nac, nasociliary nerve; V1, ophthalmic division; V2, maxillary division; V3, mandibular division. Scale bars are 10 mm. [file JOA-9999-0-s002.jpg]
